# From Artisan Experience to Scientific Elucidation: Preparation Processes, Microbial Diversity, and Food Applications of Chinese Traditional Fermentation Starters (Qu)

**DOI:** 10.3390/foods14223814

**Published:** 2025-11-07

**Authors:** Dandan Song, Xian Zhong, Yashuai Wu, Jiaqi Guo, Lulu Song, Liang Yang

**Affiliations:** 1 School of Brewing Engineering, Moutai Institute, Renhuai 564501, China; songdd0330@163.com (D.S.); zhongxian2025@163.com (X.Z.); songlulu@mtxy.edu.cn (L.S.); 2 School of Food Science and Engineering, South China University of Technology, Guangzhou 510640, China; wyss995418706@163.com; 3 China Food Flavor and Nutrition Health Innovation Center, Beijing Technology and Business University, Beijing 100048, China; guojiaq7@163.com

**Keywords:** traditional fermentation qu, qu making processes, microbial diversity, temperature regime driven, food applications and safety

## Abstract

Background: Qu was the core starter of traditional Chinese fermentation and had long relied on artisan experience, which led to limited batch stability and standardization. This review organized the preparation processes, microbial diversity, and application patterns of qu in foods from experience to science perspective. Methods: This work summarized typical process parameters for daqu, xiaoqu, hongqu, wheat bran or jiangqu, douchi qu, and qu for mold curd blocks used for furu. Parameters covered raw material moisture, bed thickness, aeration or turning, drying, final moisture, and classification by peak temperature. Multi-omics evidence was used to analyze the coupling of temperature regime, community assembly, and functional differentiation. Indicators for pigment or enzyme production efficiency and safety control such as citrinin in hongqu were included. Results: Daqu showed low, medium, and high temperature regimes. Thermal history governed differences in communities and enzyme profiles and determined downstream fermentation fitness. Xiaoqu rapidly established a three-stage symbiotic network of *Rhizopus*, *Saccharomyces*, and lactic acid bacteria, which supported integrated saccharification and alcohol fermentation. Hongqu centered on *Monascus* and achieved coordinated pigment and aroma formation with toxin risk control through programmed control of temperature, humidity, and final moisture. Wheat bran or jiangqu served as an enzyme production engine for salt-tolerant fermentation, and the combined effects of heat and humidity during the qu period, aeration, and bed loading determined hydrolysis efficiency in salt. Douchi and furu mold curd blocks used thin-layer cultivation and near-saturated humidity to achieve stable mold growth and reproducible interfacial moisture. Conclusions: Parameterizing and online monitoring of key variables in qu making built a process fingerprint with peak temperature, heating rate, and moisture rebound curve at its core. Standardization and functional customization guided by temperature regime, community, and function were the key path for the transition of qu from workshop practice to industry and from experience to science. This approach provided replicable solutions for flavor consistency and safety in alcoholic beverages, sauces, vinegars, and soybean products.

## 1. Introduction

Qu was defined as a functional fermentation starter that was produced in cereal or bran substrates by mixed microorganisms including filamentous fungi, yeasts, and bacteria under solid-state conditions. Its core role was to supply both the microbial consortium that carried out fermentation and aroma formation and the enzyme system responsible for saccharification, proteolysis, and lipolysis. It therefore occupied a central position in the solid-state fermentation system in China [1]. Historical and archeological evidence indicated a long continuity of qu-centered solid-state fermentation technologies in East Asia. At the Shangshan site in Zhejiang, pottery residues revealed plant- and fungus-based brewing with rice as the main substrate and microorganisms typical of qu such as Monacus and yeasts. The chronology reached about ten thousand years before present, which suggested that cereal fermentation initiated by qu had long served as a key node in regional technological traditions and food culture structures [2,3,4].

Within a modern fermentation science framework, qu was redefined as an engineered natural bioreactor. Under solid-state conditions with controlled moisture and convective heat transfer, community assembly and succession created multi-enzyme networks for substrate conversion. These networks supplied fermentable sugars, amino acids, and fatty acids from macromolecular substrates and drove the production of ethanol and flavor compounds [5,6,7]. Major Chinese fermented categories including Baijiu, Huangjiu, vinegar, soy sauce, soybean pastes, and soy-based products all built the first principles of flavor and structure from qu as the starting point [8]. Reviews from flavor chemistry and microbial ecology jointly argued that qu served as the vehicle for optimal microbial collaboration in traditional fermented foods such as Baijiu. Its enzyme profiles and metabolic routes determined sensory trajectories and quality stability in both alcoholic and non-alcoholic products [9,10,11,12,13]. Traditional workshops had long relied on artisanal know-how to adjust qu making such as particle size of raw materials, water addition, stacking thickness, turning points, and aeration. Under open natural inoculation, batch variability and regional effects were pronounced, which hindered consistent outputs across workshops, seasons, and sites [2,10,14,15]. Over roughly the past decade [3,11,15,16], research on qu moved from experience to measurable and model-based fermentation engineering. Heat and mass transfer in SSF acted as the nexus for advances in raw material pretreatment, online process profiling, data-driven modeling, and equipment upgrades such as intelligent qu rooms with closed-loop control of temperature, humidity, and airflow. This engineering perspective provided the methodological basis for the modern redefinition of qu [17,18,19,20]. At the frontier of microbiology [21], qu was not a static set of strains but a self-organized microecology driven by temperature trajectories, moisture, and oxygen transfer [9,10,12,13,22,23]. Multi-omics studies demonstrated thermal stratification in high, medium, and low temperature types and spatiotemporal heterogeneity during preparation and storage. Core functional guilds and metabolic routes diverged accordingly [24]. Paradigms such as qu omics connected assembly rules and metabolic phenotypes with process variables, which supplied a coherent chain of evidence for functional customization, standard evaluation, and quality traceability of qu [2,3,25,26,27,28,29].

Against this background, this review aimed to address three focused research questions. The first concerned process engineering. Raw material types such as wheat, barley, pea, rice or rice flour, and bran, together with forming and drying methods, moisture and aeration settings, and stacking thickness, jointly shaped the pore structure and the regimes of heat and mass transfer in qu, which defined enzyme profiles and subsequent fermentation kinetics. The second concerned community ecology. Thermal types and environmental factors drove assembly and functional divergence of mixed microbial communities, which mapped through enzyme substrate microbe coupling to flavor chemistry routes. The third concerned food applications and quality and safety. The functional positions of different qu in alcoholic beverages, sauces, vinegars, and soy-based products, together with key control points QCP and risk factors such as *Monascus*-related mycotoxin citrinin and biogenic amines, formed a closed loop from identification to intervention. Based on these three questions, this review integrated the four dimensions of process, microecology, flavor, and quality into a testable research framework intended to support the transition from artisanal practice to industrialization and intelligent manufacturing. At the academic level, this work juxtaposed SSF engineering with community ecology and flavor omics to rebuild the evidence chain from variables and mechanisms to phenotypes and to provide material for causal models linking qu functions, fermentation performance, and product flavor. At the industrial level, this work focused on implementable parameters and indicator systems such as moisture and aeration-linked temperature trajectories, enzyme intensities, yields of flavor precursors, and sensory readouts for standardized qu making, cross-site consistency, and development of greener products with reduced salt and sugar.

To ensure traceable scope and evidence, databases included Web of Science Core Collection, Scopus, PubMed, and Google Scholar for historical and technological sources. The time window covered 2000 to October 2025. English peer-reviewed papers and authoritative reviews were prioritized. The keyword strategy combined subject headings and free terms. The terms included Jiuqu, Qu starter, Daqu, Xiaoqu, Hongqu, *Monascus* or koji Chinese, soy sauce koji, rice wine starter, together with microbiome, metagenomics, process or engineering, and solid-state fermentation. Chinese terms were used in parallel for cross-checking of history and terminology. Title and abstract relevance guided the first screening. Inclusion in the second screening required explicit process parameters, community data, or flavor or safety indicators. Duplicates were removed before thematic synthesis. To avoid narratives without data, verifiable parameters or quantitative ranges were reported at key assertions. Objects, methods, and sources of uncertainty were specified when cross-study comparisons were made.

## 2. Process Engineering of Qu Making

### 2.1. Daqu from Temperature Regime to Finished Starter Process Engineering

**Figure 1:**
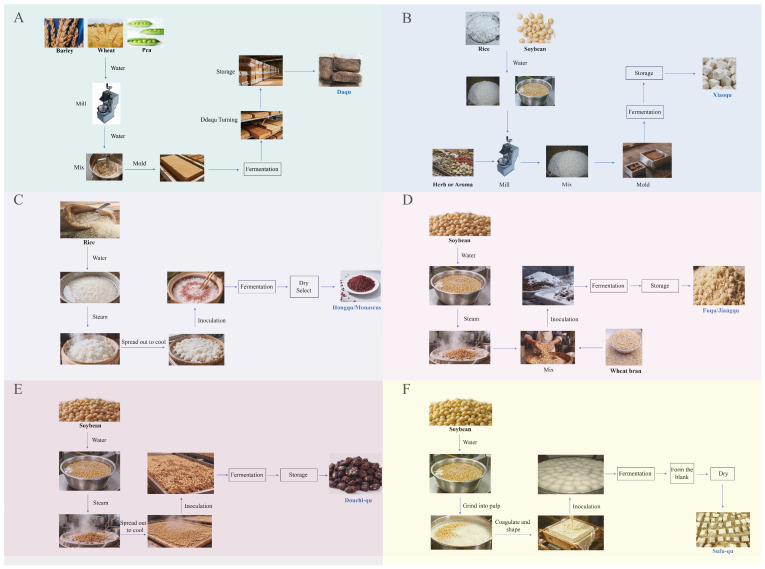
Schematic of qu-making processes and process engineering. Panels (**A**–**F**) were daqu, xiaoqu, hongqu (*Monascus*), fuqu/jiangqu, and sufu-qu.

Daqu preparation was viewed as an open solid-state fermentation chain that linked raw materials, forming, heap cultivation, drying with maturation, and storage. The core control variables were not isolated recipes (Figure 1A). They were boundaries of heat and mass transfer defined by brick geometry and stacking and the coupled temperature–moisture trajectory that evolved on these boundaries. The traditional low, medium, and high temperature types defined by the maximum core temperature were more than quality labels. They corresponded to distinct thermal histories and moisture paths, which determined enzyme retention, precursor formation, and the kinetic fitness of downstream fermentation [30]. Within this framework, selecting a temperature type amounted to a combined setting of target heat flux, evaporation flux, and oxygen transfer capacity. The engineering meaning exceeded the fine-tuning of any single parameter.

During heap cultivation, the rise, hold, and fall of temperature were dominated by biological heat. Ventilation and surface evaporation shaped the peak and its decline. Rather than listing exact times, emphasis was placed on coordinated scheduling of turning, aeration, and humidification and its role in within-batch consistency [31]. Turning reshaped vertical gradients of heat and moisture, synchronized drying and maturation among positions, and reduced early enzyme inactivation caused by local overheating or over drying. Programmed control of airflow and moisture stabilized peak level and rewetting rhythm within a given temperature window, which provided a reproducible thermal background for target enzyme profiles and flavor precursors [31]. Recent mappings of spatiotemporal profiles showed that the middle layer often carried higher moisture and gentler fluctuations. This heterogeneity arose from mismatches among stacking density, porosity, and duct layout. It was more effective to co-optimize heap geometry and air paths at the design stage to smooth potential gradients in advance, so that top, middle, and bottom layers experienced similar drying and maturation, which reduced batch and layer variation at the source [32]. Under the same formula, stacking geometry and airflow organization often became the watershed for obtaining good qu because they changed effective heat exchange area and gas–solid contact, and in turn reshaped temperature–moisture trajectories and metabolic flux.

Initial moisture and forming parameters showed threshold effects on early heating and structural stability [30]. Excessive moisture suppressed early heating and increased collapse and sticking. Insufficient moisture led to weak heating and early enzyme deactivation. It was more meaningful to discuss these variables within the feasible region defined by achievable temperature type and controllable peak shape rather than fixating on a single value. Brick size and density determined conductive paths and flow resistance. Standardized geometry provided reproducible heating and drying under similar climates. Pursuit of higher peak temperature or a richer volatile profile required a new balance between stacking density and ventilation intensity to avoid overfiring or overdrying that harmed enzymes and precursors [30]. Cross-workshop harmonization should not rely on a universal moisture or brick-weight threshold. Combinations of heap form, ventilation, and humidification were back-calculated from the target temperature type and ambient climate [4,33,34].

Online monitoring and closed-loop control were necessary to make the temperature–moisture trajectory explicit and parameterized [35]. Drying and maturation inherited water and heat management from the heap stage. The task was to obtain mechanical and transfer stability suitable for storage and dosing without sacrificing enzymatic activity. Rather than emphasizing a single terminal moisture or a fixed drying temperature, attention was paid to the coupling among drying rate, stress release of the body, and storage time. Rapid water loss caused structural stress and surface densification, which impaired later water uptake, disintegration, and uniform dosing. Slow drying increased self-heating and secondary rewetting risk. Plants therefore used airflow organization, tiered relocation, and split sampling to align drying rate and storage duration with the post-treatment window of the selected temperature type, which promoted within-batch uniformity and cross-batch comparability [10,36].

In sum, finished quality was governed by the four-factor coupling of temperature type, heap form, ventilation, and moisture, while external climate expanded or narrowed the feasible region by altering dissipation and evaporation boundaries [37]. Cross-season and cross-site consistency depended on reconstructing boundary conditions and process curves according to climate rather than locking a fixed recipe so that target peak temperature, heating slope, and rewetting window remained reproducible and comparable [37]. This path agreed with recent studies linking temperature and precipitation to differences in community assembly and metabolism of daqu, which suggested a stable mapping between engineering variables and microbial ecology that supported process design and quality prediction.

### 2.2. Xiaoqu Engineering Control of the Rice Based Dual Fermentation Platform

The engineering essence of xiaoqu was framed as a continuous chain that used rice or rice flour as the carrier and linked forming, short incubation, mild drying, and stable storage to build a solid inoculum with long shelf life and controllable dosing (Figure 1B). Unlike daqu that relied on thick heaps and metabolic heat, the thermal and moisture trajectory of xiaoqu was governed by room boundaries. Key variables concentrated on dough moisture, piece size, and the coupling of ventilation with dehumidification. These parameters determined the early exothermic curve, the retention of the enzyme profile, and the handover efficiency of the dual fermentation in which saccharification preceded alcohol formation [30]. During the forming and incubation of the front half, moisture and shaping were more critical than any single operating detail. Higher dough moisture created a continuous aqueous phase and cohesive structure, which supported early heat release and stable mass transfer. Miniaturized pieces lowered internal thermal resistance so the rise and fall of product temperature could be finely controlled by room temperature, humidity, and air exchange. Their coupling set the starting activity, within-batch consistency, and the repeatability of the drying endpoint. Modern descriptions therefore emphasized inoculation with standardized mother powder and consistency management of forming so that the short thermal history of heat up, hold, and cool down remained reproducible [38].

During incubation, thin spreading with medium temperature and high humidity served as the dominant strategy to obtain a stable thermal history. Thin layers reduced vertical gradients, medium temperature limited early enzyme deactivation, and high humidity prevented a dry crust while supporting continuous mycelial expansion. Rather than listing exact set points, control prioritized their relative weights. When air exchange was insufficient, thinning the layer and staged ventilation were used to dissipate heat accumulation. When surface water loss increased, gentle humidification and covering were used to maintain the interfacial water film and avoid an irreversible barrier layer. This combination enabled xiaoqu to develop a reproducible short thermal history within days and to deposit a stable enzyme profile, which preserved process flexibility for storage and dosing [39,40,41,42]. Related studies on xiaoqu in rice wine and regional Baijiu likewise indicated that coordinated optimization of room temperature, humidity, and ventilation determined the predictability of aroma and ethanol formation paths. In drying and curing, the focus lay in balancing activity retention, storage safety, and mechanical stability rather than chasing a single endpoint. Mild hot air or natural ventilation aimed to yield low moisture, structural stability, and easy disintegration and rehydration at dosing. Subsequent room temperature dark storage was used to release stress and stabilize physicochemical and sensory precursors. Although final moisture thresholds were often used as a general safety line, the engineering emphasis rested on the coupling among drying rate, internal structure, and activity decay. Overfast drying caused surface densification and hindered internal migration, whereas overslow drying raised the risks of rewetting and contamination. Joint release that considered final moisture, block strength, and disintegration was preferable to any single indicator [43,44,45,46]. Systematic reviews of rice wine processing likewise highlighted that the advantages of xiaoqu in storage stability and downstream consistency arose from parameterized management at the drying endpoint and during curing rather than from a fixed operating number.

As the interface of the rice-based dual fermentation, xiaoqu also paced dosing. Instead of enumerating numerical ratios or temperatures, two matches were stressed. The substrate temperature before dosing needed to return to a medium range to reduce thermal stress and protect the enzyme system. The dosing ratio needed to align with the degree of cooking and bed thickness so that the kinetics of saccharification in the front half and alcohol formation in the back half remained matched, and phase mismatch in sugar to alcohol conversion was avoided. This coordination of temperature, dose, and loading was key to strong initiation, steady conversion, and predictable flavor, and it matched the engineering features of short room temperature incubation and mild drying in xiaoqu to form a continuous and controllable chain from inoculation to main fermentation [42,45].

For quality control and standardization, window type indicators were preferred over rigid single points at process endpoints. An upper window of qu acidity was used to judge whether moisture loss and acid accumulation during incubation were acceptable, and it was combined with final moisture, block strength, and disintegration for release. On the equipment side, fixed diameter and weight molds, zoned ventilation, and online temperature humidity monitoring reduced within-batch thermal and moisture gradients and improved consistency across seasons and facilities [42,47].

### 2.3. Hongqu (Hongqu/Monascus) Dual-Objective Engineering for Pigment and Aroma Production with Toxin Control

Hongqu was prepared on steamed rice as a solid-state koji. The process was framed as a compromise that maximized color and aroma while minimizing the risk of CIT within coupled boundaries of moisture, temperature, oxygen supply, and heat dissipation. Rather than listing fixed settings, these variables were treated as factors of one objective function (Figure 1C). Initial moisture and ambient relative humidity defined resistance to heat and mass transfer and also governed oxygen diffusion. The temperature program and aeration reshaped the profile of metabolic heat and the window for pigment synthesis. Practice indicated that a variable temperature profile with moderate moisture increased product activity and suppressed toxin accumulation. The reproducibility of the thermal and moisture trajectory was more critical than a single set point. A representative study achieved “not detected” CIT by staged temperature control with moderate moisture, which exemplified optimization through process windows instead of point parameters [48]. Within this framework, excessive moisture caused agglomeration, reduced effective surface area, and hindered oxygen diffusion, which depressed coloration and created abnormal hot spots. Insufficient moisture limited metabolism and pigment formation. The temperature zone also showed an optimum band. Early overheating raised self-heating risk and induced by-metabolism, whereas overcooling delayed growth and synthesis. Programmed staging with gentle drying stabilized the metabolic route [49].

Equipment and bed design were aligned to uniform oxygen delivery with controlled heat removal and dehumidification. Compared with thin bamboo trays, perforated beds and rotary or recirculating-air devices shortened the initial lag and smoothed interlayer gradients. Batches then reached more consistent color and activity. These systems shaped the metabolic thermal history through bed homogenization and programmable temperature–humidity curves, which reduced intervention while stabilizing output [50]. Regardless of carrier or duct layout, the core objective was to reduce spatial stratification that produced hot interiors and cool exteriors and to hold parameter fluctuations within the target window so that process consistency yielded quality consistency.

Quality and compliance were integrated into one control loop. In Europe, products based on red yeast rice were controlled at a maximum level of 100 µg/kg for CIT, and market surveys reported exceedances. Production therefore linked parameterized pretreatment, bed heat–moisture management, programmed cooling, early mild drying, and endpoint release testing as one strategy. Source suppression was implemented in parallel with terminal quantification by HPLC or LC–MS/MS, which avoided reliance on a single end step [51]. Drying and deactivation were managed under a triple objective of stability, safety, and functional retention. Mild hot air and timely deactivation preserved pigments and functional components and enabled low-moisture storage. Source control along the chain was more feasible and cost-effective than post-process removal [52].

### 2.4. Fuqu/Jiangqu the Enzyme Engine for Salt-Tolerant Fermentation

Fuqu/Jiangqu used a composite matrix of wheat flour or bran with soybean or defatted soybean meal. The engineering aim was to build an enzyme engine that still worked efficiently during subsequent high-salt fermentation. The matrix ratio determined porosity and apparent moisture. It reshaped heat and mass transfer boundaries and the aw window during the koji stage (Figure 1D). Under the same temperature, humidity, and aeration, a bean-rich formula, a bean–wheat parity formula, and a bran-as-skeleton formula were not equivalent in enzyme profiles or salt-stage hydrolysis efficiency. These differences amplified regional process variations in flavor and in the release rate of amino nitrogen [53]. In this framework, the primary role of thermal treatment was not edibility. It lowered contaminant loads and stabilized microstructure, which provided an adherent, oxygen-permeable, and moisture-drainable base for *Aspergillus*. Thus, raw material histology and room climate acted as coupled variables that jointly determined enzyme trajectories rather than independent factors [53].

Inoculation and bed loading were treated as decisive parameters. Compared with dry sprinkling, liquid starters with metered spraying or rolling coatings improved spatial uniformity and synchrony of awakening. Bed positions then approached a shared thermal and moisture history, which reduced turning burden and early enzyme decline. Liquid inoculation validated gains in structure, pH, and key hydrolase activities and supported advantages of optimized bean to wheat ratios at the enzyme output, providing a reference for cost reduction and efficiency improvement on the raw material side of fuqu [54,55]. From bed geometry to oxygen supply paths, flat thin beds, multi-layer conveyors, and perforated rotary beds shared one goal. They maintained boundaries that allowed heat removal, moisture discharge, and uniform oxygen supply at the bed scale. Thickness switching and zoned ventilation functioned as process tools to offset metabolic heat peaks and local hypoxia. Their performance finally appeared in color, odor, and enzyme protein stability within the same batch [55,56,57].

Control of temperature, humidity, and aeration shifted from set points to trajectories. Static settings of a single temperature or relative humidity could not satisfy germination, hyphal spread, and secretion phases at once. A dynamic coupling of temperature control, moisture retention, oxygen supply, and heat removal used time-segmented turning and aeration to manage heat peaks and to dissolve interlayer gradients. This supported spatially uniform maturation and temporally stable enzyme production. The direct benefit was stable salt-stage hydrolysis and predictable flavor precursors without extra enzyme addition [56,57]. Release criteria at the end of koji making were also shifted. Empirical judgments based on color and hand feel were weakened. Structural integrity, moisture, and functional enzyme activities formed composite thresholds for release as auditable and reproducible control points [58].

The final test occurred after salting. High salt marsh or low-salt solid systems inhibited *Aspergillus* growth, yet the extracellular enzymes formed and anchored in the solid phase during the koji stage carried the main hydrolytic task. Each upstream adjustment of moisture, heap geometry, aeration, and turning was magnified through a chain of porosity, heat generation, enzyme profile, and salt-stage stability. It then appeared as differences in the formation rates of soluble amino nitrogen and reducing sugars and in flavor layering of moromi. Treating formulation, bed loading, oxygen supply, and heat removal as an integrated process design rather than a disconnected checklist was the key path by which fuqu/jiangqu moved from artisanal experience to industrial consistency [53,54,55,56,57,58].

### 2.5. Douchi-Qu: Process Branches of Multi-Type Starters

The qu making for douchi was the first half of a two-stage process. The goal was not a fixed set of optimal parameters. The goal was to build a stable and scalable starter on cooked soybean or black soybean surfaces. This starter provided a reproducible enzymatic base and a physical interface for later salting and maturation. From a process view, preprocessing, inoculation, and qu formation formed a continuum of heat and mass transfer with community assembly (Figure 1E). Raw material cooking set pore structure and bound water. Cooling and inoculation set initial colonization and contamination risk. Bed arrangement and room climate set the coupling efficiency of metabolic heat release and transfer paths. It was more useful to identify sensitive variables, their interactions, and the trade-offs across process branches than to list formulas, times, and thicknesses [59].

Moisture, aeration, and bed thickness acted as the main coupled factors during qu formation. Excess kernel moisture weakened oxygen diffusion and enlarged heat accumulation. Insufficient moisture limited hyphal front advance and extracellular enzyme secretion. Poor aeration caused an inner hot and outer cool gradient. Excess aeration caused dry crust and local desiccation. A very thick bed increased vertical thermal resistance. A thin bed improved heat removal and vapor migration but required stricter control of airflow uniformity and floor space. Practice showed that better outcomes were achieved by moderate moisture with directed aeration and a medium bed to balance the heat and moisture field. This setting supported continuous oxic metabolism and avoided overlapping of peak temperature and rewetting risk in space. Window ranges rather than point values reduced batch and seasonal effects and eased scale transfer [59].

Three main process branches were present. Their differences lay in rhythm, boundary conditions, and endpoint control. The *Aspergillus*-led route used a fast rhythm and higher process controllability. Stable room temperature and humidity with uniform spreading rapidly built hyphal networks and multi-enzyme systems. It supported clear release criteria and smooth transfer to salting. The cycle was short and scalable, but it relied more on uniform cooling and inoculation [60]. The *Mucor* or *Rhizopus* route emphasized thin layers and zoned aeration to relieve interlayer heat build-up and to keep controllable water gradients. The cultivation period was longer and often used mild washing and pre-drying to stabilize the qu–salt interface moisture. It gave typical texture shaping and flavor precursors and was more sensitive to airflow organization and final drying strategy [61]. The bacteria-led or bottled solid route changed container scale and thermal resistance to gain a more uniform heat and moisture field with lower contamination risk. It suited standardized and clean production. It needed container geometry and loading design to compensate for the loss of environmental diversity seen in open beds. It improved uniformity while weakening environmental selection pressure [62]. Comparative studies showed repeatable differences in time constants. *Aspergillus* and *Rhizopus* routes ran on tighter rhythms. *Mucor* routes required a longer enzyme-building stage. This pattern supported planning and equipment use without fixing a single duration [63]. Temperature trajectories described engineering quality better than single points. Many flows used a moderate initial rise, a stable platform, then a slow decline to match hyphal expansion and extracellular enzyme peaks. Control focused on the rate of rise and platform stability to avoid core overheating and on a gentle final decline to promote water redistribution and reduce crusting. The *Aspergillus* route favored a stable platform for sustained secretion. The *Mucor* or *Rhizopus* route was more sensitive to the heating gradient and airflow direction. Bottled processes relied on both environment and container control to achieve equivalent management. Trajectory design was prioritized over absolute values [59,60,61,62].

Release endpoints and interface moisture set salt diffusion rate and osmotic shock in the salting stage. Overwet endpoints caused local dilution and osmotic oscillation at the start of brining. Overdry endpoints inhibited brine penetration and affected precursor migration and conversion. Each route used short pre-drying, warm water washing, or natural re-softening. The aim was not a single moisture number. The aim was to place qu moisture and surface state within a reproducible interface range. Stable salt diffusion, proteolysis, and flavor release kinetics were then obtained. This approach emphasized endpoint ranges. It allowed predictable maturation under different seasons and equipment conditions [60,61].

In sum, process design for multi-type douchi starters began from variable sensitivity and coupling. A ternary set of moisture, aeration, and thickness shaped a controllable heat and moisture field. Temperature trajectories rather than point values aligned hyphal expansion and enzyme profiles. Endpoint ranges rather than a single moisture value secured seamless handover to salting. Within this frame, different branches balanced cycle time, uniformity, and flavor potential [59,60,61,62,63].

### 2.6. Sufu Qu Preconditioning Engineering for Cheese Like Texture

Sufu-qu functioned as the key transition from a tofu matrix to a controllable mold curd and established a stable entry point for the enzyme–texture interface required by salting and ripening. The aim of the pretreatment was to tune substrate moisture, pore structure, and initial microbial load to support surface growth of molds while keeping internal integrity (Figure 1F). Common coagulants such as bittern or gypsum with moderate pressing created a balance between high moisture and connected pores, which afforded adhesion and spreading of the mycelial layer and penetration of extracellular enzymes. Standardized heat treatment and inoculation reduced interference from undesirable microbes and improved within-batch consistency. These factors jointly determined the uniformity of mold coverage and the reproducibility of the enzyme system during the qu-making stage [64,65].

Room cultivation was governed by coupled optimization of temperature, humidity, and time. Moderate temperature and near-saturation humidity provided a permissive range for white-mold sufu to grow and secrete enzymes, yet strains responded differently within this range. Lower temperature with a moderate cultivation period favored a continuous, compact mycelial layer and balanced surface water loss with internal migration. Higher temperature or shorter cultivation can raise single-enzyme activities but tended to cause surface crusting or internal heat build-up, which enlarged within-batch variation. Evidence indicated that the preferred window was defined by stable output of combined protease, lipase, and amylase rather than a peak of one enzyme, which protected mold quality and the stability of later texture formation [66].

Tray geometry and airflow organization determined spatial uniformity of oxygen supply and heat release. Thin-layer loading, spaced stacking, and zoned supply–return air managed evaporative cooling and removal of metabolic heat at the bed scale and reduced thermal stratification and end cracking. Matching inoculation with loading strategies shortened awakening heterogeneity and cut the need for frequent turning. Process monitoring of gap size and face velocity helped maintain similar thermal–moisture trajectories across batch sizes and equipment and achieved spatial consistency of thermal history in the same chamber [67,68,69].

Release criteria and interface moisture were anchored to whether salting could proceed stably while preserving texture–flavor synergy. Fingerprint-style indices were preferred over a single endpoint. Coverage and continuity of the mold layer, stable ranges of moisture and soluble solids, and mechanical integrity with predictable salt uptake served as the basis for release. For products requiring a denser surface and a finer cheese-like mouthfeel, a lower temperature with a modestly extended cultivation was used while maintaining high humidity and avoiding excessive convection to keep interface continuity. In summary, a method chain that linked tofu substrates of fixed size and loading, a short moderate-temperature high-humidity window, harmonized tray airflow, and release standards centered on functional enzyme spectra, and texture intake was adopted. This approach enabled reproducible sufu molds in non-aseptic and scaled environments and provided a benchmarked pretreatment interface for subsequent salting and flavor maturation [65,70].

## 3. Microbial Diversity and Community Ecology

### 3.1. Daqu Communities, Temperature-Regime Driven Assembly, and Functional Differentiation

Daqu was an open solid-state composite inoculum. Community assembly was strongly driven by the temperature regime, defined by the highest temperature reached during making. By peak temperature it was grouped as low temperature at about 40–50 °C, medium temperature at about 50–60 °C, and high temperature at about 60–70 °C. This stratification matched distinct thermal histories during making and systematic differences in community composition and functional outputs. A representative study on strong-aroma daqu tracked the full course from making to storage, defined these temperature zones, and compared co-evolution of community structure and physicochemical indices [71].

The primary driver role of temperature appeared in quantitative shifts in alpha and beta diversity and in replacement and enrichment of core taxa. For medium-temperature daqu (MT-Daqu), mature inner and outer layers reached 625–773 and 597–632 OTUs, respectively, by 16S or ITS sequencing, showing a spatial heterogeneity with higher richness at the center. At the genus level, *Bacillus* dominated bacteria, with 47.02% relative abundance in the inner layer versus 21.07% in the outer layer, while *Saccharopolyspora* reached 6.11% in the outer layer versus 0.79% in the inner layer. Counts of aerobes and yeasts dipped when the temperature rose to about 60 °C on day 17, then recovered during storage and stabilized after three months. This indicated coupling between the heat peak, slow cooling, and maturation and the restructuring of the community [71].

High-temperature daqu (HT-Daqu) under stronger heat stress showed a dual dominance by thermotolerant bacteria and fungi. A regional comparison across four HT-Daqu samples from core producing areas in northern Guizhou and adjacent Sichuan and Chongqing found *Desmospora* sp. 8437 as a consistent dominant taxon at 3.6–7.3% by metagenomics. *Bacillaceae* correlated positively with differences in saccharifying and proteolytic power and with pyrazine levels. *Kroppenstedtia* associated with variations in aldehydes and ketones, revealing redundancy and division of labor among functional cores under similar thermal histories. This work also noted production peaks up to 60–75 °C in HT-Daqu, supporting the chain of high temperature, thermotolerant communities, and strong pyrazine- and protein-cleaving capacity [72]. At finer time scales, heat pulses promoted a phylum-level shift toward Firmicutes. Parallel monitoring of sauce-aroma HT-Daqu made by hand or machine showed that, over 40 days, Firmicutes rose from 43.15% and 27.16% to 93.10% and 97.88%. Proteobacteria fell from 41.13% and 54.26% to 6.53% and 1.50%. At the genus level, Lentibacillus rose to 72.19% and 40.16% by day 40. This showed that spore-forming, salt- and heat-tolerant bacilli gained overwhelming advantages in the high-temperature window. The differences indicated that even with different forming methods the thermal history remained the first cause deciding the core taxa [73].

In contrast, low-temperature daqu (LT-Daqu) showed a yeast–lactic acid bacterium cohabiting signature in the initial community. In a multi-omics study of light-aroma systems, mature daqu contained Ascomycota at about 39%. At the genus or species level, *Pichia* at 33.2% and *Lactobacillus* at 17.4% were dominant. At the start, *Pichia* and *Lactobacillus* were 24.0% and 20.7%. Yeasts then declined while lactic acid bacteria increased as fermentation progressed. This reflected a mild thermal history with rising acid stress and takeover by LAB. The pattern matched the lower peak temperature of LT-Daqu and explained a gentle co-platform for saccharification and acid production [74].

Different temperatures changed what was present and what each group did. In MT-Daqu, the dominant filamentous fungi split by position. *Aspergillus* and *Rhizopus* were enriched inside. *Thermomyces*, *Thermoascus*, and *Rhizomucor* were enriched at the surface. On the bacterial side, *Bacillus* dominated inside and *Saccharopolyspora* at the surface. This pointed to inner-layer priming of starch and protein hydrolysis, surface-layer dominance in thermotolerant enzymes, lipolysis, and conversion of volatile precursors. In HT-Daqu with color types, bacterial alpha diversity by Chao and Shannon showed a dip followed by a rise in maturity, and dominant genera differed among colors. Black qu was dominated by *Kroppenstedtia*, which rose to 68.20% on day 30 then fell to 3.11% on day 180. Yellow qu favored *Virgibacillus*. White qu kept *Bacillus* above 80% and maintained higher fermentative activity. Fungi were commonly centered on *Thermoascus*. These results showed that heat history and acidity reshaped the functional core by selection pressures and that new steady states formed during maturation and storage [30,71].

Taken together, temperature regime, community assembly, and functional differentiation followed three representative ecological routes. First, LT-Daqu formed a mild community at lower peaks with yeast and LAB synergy that supported early coupling of saccharification and acid production. Second, MT-Daqu formed an inner–outer division of labor under moderate peaks and higher inner alpha diversity, with *Bacillus* plus filamentous fungi such as *Aspergillus* or *Rhizopus* inside versus *Thermomyces* or *Thermoascus* at the surface. Third, HT-Daqu under high heat pulses and extended holds selected thermotolerant core bacteria such as *Bacillaceae*, *Kroppenstedtia*, and *Desmospora*, which coexisted with thermotolerant fungi such as *Thermoascus*. Community function shifted toward efficient cleavage of proteins and polysaccharides and formation of nitrogen-heterocycle flavor precursors. Multi-source sequencing and metagenomic quantification including relative abundance, OTU or ASV counts, alpha diversity indices, and time–space comparisons supported these conclusions and reflected a temperature-history-centered ecological rule in Chinese solid-state fermentation.

### 3.2. Xiaoqu Communities, a Rhizopus–Saccharomyces–Lactic Acid Bacterium Symbiotic Network

Compared with daqu, rice-based xiaoqu showed fewer core genera with more pronounced dominance. This allowed rapid formation of a functional network based on molds, yeasts, and LAB early in fermentation. A comparison of Cantonese fire-aroma or chi-aroma and rice-aroma systems reported fewer species in xiaoqu than in daqu. *Rhizopus*, *Saccharomyces* and related yeasts, and LAB still supported integrated saccharification and alcohol fermentation. This indicated a small but efficient configuration as the ecological background [75].

In rice-aroma baijiu, the fungal side showed dual dominance of molds and yeasts. At the genus level, *Saccharomyces* reached 7.06–83.50% and *Rhizopus* 15.21–90.89%. In xiaoqu itself, *Saccharomyces* once reached 88.41%. After inoculation, *Rhizopus* rose rapidly from 15.21% and peaked at 90.89% on day 10. This formed a relay with molds driving saccharification and yeasts producing alcohol. On the bacterial side, LAB dominated. Across xiaoqu, saccharification, and fermentation, *Lactobacillus* reached 62.88–99.23% and reached 99.23% at the end. In xiaoqu itself, the top four bacteria were *Weissella* at 59.53%, *Pediococcus* at 29.18%, *Acetobacter* at 3.65%, and *Lactobacillus* at 1.35%. This showed an assembly that opened with *Weissella* and *Pediococcus* in the starter and then shifted to *Lactobacillus* after vatting [76]. Multi-omics work also revealed the source and assembly logic. In a rice-aroma system, xiaoqu held 383 bacterial species yet shared only 48 with day-0 mash and only 10 with the end of fermentation. In contrast, xiaoqu held only five fungal species. Xiaoqu therefore provided the core framework with *Rhizopus* and *Saccharomyces*, while many bacteria entered later from raw materials and the environment and were reshaped under acid stress as LAB rose. Dominance was highly concentrated. During rice-aroma fermentation, the top five bacterial genera, *Lactobacillus*, *Weissella*, *Pediococcus*, *Lactococcus*, and *Acetobacter*, exceeded 97%, giving the community a strong acidifying tendency [76].

Cross-system evidence reinforced a three-stage symbiosis of *Rhizopus* first, yeast takeover, and LAB steady state. In parallel monitoring of hongqu rice wine and xiaoqu rice wine, *Rhizopus* in xiaoqu reached 32.14% in the starter, jumped to 80.69% on day 2, and stabilized near 50% mid-fermentation. This matched the rising trend of *Rhizopus* in rice-aroma baijiu and pointed to a shared path of rice substrate, mold saccharification, and acid-mediated competition. Late dominance by *Lactobacillus* near 99% coincided with accumulation of ethyl lactate. In rice-aroma systems, ethyl lactate often exceeded ethyl acetate, and 2-phenylethanol could reach more than ten times the sum of those two. This reflected coupling between lactic acid, ethanol, and ethyl lactate driven by LAB and yeasts [76,77].

From a coupling view, metabolism in xiaoqu communities focused on energy and structural substrates. An integrated metagenome and metabolome study of traditional Guizhou xiaoqu showed that metabolic functions accounted for 58.16% of microbial genes, followed by genetic information processing at 12.57% and organismal systems at 8.29%. Carbohydrate metabolism, cofactors and vitamins, and amino acid metabolism were the most enriched pathways. This matched the division of labor in which *Rhizopus* supplied sugars, yeasts produced alcohol, and LAB shaped an acid environment and drove ester precursors. *Rhizopus*–*Saccharomyces*–LAB was thus not a simple inoculation trio. In a rice-based solid environment, it formed a self-organized network through substrate release, control of pH and redox, and selection pressures. Core quantitative features including genus-level abundance ranges, assembly overlap, and functional enrichment showed consistency across independent lines of study [78].

### 3.3. Hongqu Communities, Monascus-Centered Secondary-Metabolism Ecology

Rice-based hongqu, also known as red koji or red yeast rice, was a fungal-led and multi-domain solid micro-ecosystem centered on *Monascus*. *Monascus* led early fungal establishment at the community level and determined pigment, lovastatin, and mycotoxin potential such as citrinin through multiple biosynthetic gene clusters. In practical dynamics, *Monascus* often dominated at the start after inoculation. *Saccharomyces* then became dominant after day 3. *Aspergillus* could also co-dominate with yeasts in some systems and in comparisons of xiaoqu rice wines and hongqu or huangjiu. This *Monascus*-first and yeast-takeover trajectory was repeatedly observed in metagenomic studies of hongqu rice wine [79,80].

Joint metagenomic and metabolomic analyses depicted *Monascus*-driven coupling. In a comparison of high- and low-biogenic-amine systems for hongqu rice wine, *Monascus*, *Saccharomyces*, and *Aspergillus* were the three core fungal genera. In high-amine systems, *Monascus* dominated early and *Saccharomyces* became absolutely dominant after day 3. In low-amine systems, *Aspergillus* plus *Saccharomyces* co-dominated and *Monascus* declined. Species–metabolite networks showed significant associations between dominant fungi and volatiles or amines derived from amino acid metabolism. This suggested that *Monascus* shaped the substrate framework and pH or redox through pigments, organic acids, and polyketides in the early stage, which created conditions for later takeover by yeasts and LAB [79].

Community and metabolism also differed by regional hongqu types. In two widely used types, Gutian qu and Wuyi qu, *Monascus* species dominated in the former. The two types showed distinct pathway enrichments for fungal communities and flavor formation during fermentation, indicating that the *Monascus*-centered niche varied in strength and direction among types. These differences were also reflected in enrichment of downstream metabolic routes such as amino-acid-derived volatiles and in abundance networks of key enzyme genes [81].

*Monascus* secondary-metabolism ecology was not single-track. Potential for monacolin K varied greatly among species and strains. An industrial M. pilosus reached 9.5 mg/g MK under optimized solid-state conditions with no CIT detected, while some *M. purpureus* lacked MK loci. In co-culture with yeasts or LAB, reports noted pigment up-regulation with toxin down-regulation or broader profile shifts. This indicated a self-organized system modulated by interaction signals such as fatty acids and pH. Cross-level indicators including gene cluster length and composition, gene counts, time nodes, and product concentrations supported this picture. Through its BGC architecture and expression program, *Monascus* set energy and carbon flows in hongqu communities and guided time-scale rearrangement and stabilization across sugar, amino acid, and polyketide networks.

### 3.4. Fuqu and Jiangqu Communities, a Relay Between Koji-Stage Molds and Salt-Stage Moromi Bacteria

Fuqu or jiangqu systems showed a two-stage ecological relay. The first stage was the koji period on bran–soy or wheat substrates. The second stage was the high-salt dilute moromi. In the solid and oxygen-rich koji stage, filamentous fungi and facultative salt-tolerant bacteria expanded rapidly and assembled a high-throughput hydrolytic enzyme pool. After transfer into moromi at 18–22% NaCl, the hypertonic, low-oxygen liquid selected salt-tolerant LAB and halophilic yeasts. Diversity converged over time on a few core groups. The ecological task shifted from enzymatic breakdown to salt-phase conversion. Under typical practice, the koji stage lasts about 26 h to 7 days. Moromi at 18–22% NaCl lasted about 3–6 months [56,57]. During koji, the relative abundance of molds rose together with some early colonizing bacteria. In samples from industrial high-salt diluted soy sauce, *Aspergillus* and *Weissella* rose from 0.98% and 0.31% to 38.45% and 30.41% within 0–48 h. Amino acids and volatile precursors accumulated markedly, providing substrates and exogenous enzymes for moromi. The study also noted that a solid, well-ventilated, and high-humidity room near 95% supported coexistence of fungi and facultative salt-tolerant LAB, which anticipated the later salt-stage relay [56].

After mixing koji with brine, salinity and oxygen transfer became decisive filters. In a metagenomic comparison of Japanese-style and Cantonese-style moromi, Tetragenococcus rose from 0.02% to 59.2% between days 7 and 120 in the Japanese style. In the Cantonese style at 120 days, Tetragenococcus and Staphylococcus reached 36.7% and 29.7%. Koji-borne fungi rapidly moved to the margins in moromi. *Aspergillus* averaged up to 18.2% early in the Japanese style or 10.93% in the Cantonese style but fell to 0.65% or less by the end. At the family or phylum level, Aspergillaceae signals dropped from 33.0% and 17.5% to 0.13% and 0.67%, while the whole community shifted toward bacteria. Bacterial sequences rose from 59.3% to 98.5% in the Japanese style. These data pointed to high salt suppressing continued growth of koji-borne molds and opening niches for salt-tolerant LAB and cocci that drove late acidification and nitrogen- and carbon-backbone conversions [82]. The physiology behind this relay matched salt-tolerance limits. Tetragenococcus halophilus grew at up to about 20% NaCl and showed an optimal zone often at 5–10% yet retained activity at higher salinities, which matched 18–22% NaCl in moromi and gave it a competitive advantage to control organic acid and amino acid routes [83,84]. The osmophilic and halotolerant yeast *Zygosaccharomyces rouxii* also adapted at 18% NaCl and contributed to flavor metabolism. Transcriptomic and lipidomic data showed higher unsaturated fatty acids under salt and formation of key flavor precursors such as 3-methyl-1-butanol and 2-phenylethanol [85,86,87,88]. Dominant taxa in moromi were not fixed. They were coupled with temperature and aeration strategies. With the same koji, Japanese-style practice near 25 °C with some stirring favored Tetragenococcus and *Weissella*. Cantonese-style practice at 18–22 °C with little stirring often favored Staphylococcus and higher *Bacillus* signals. This temperature–oxygen coupling explained metabolic and flavor differences among process families at the same salinity and again verified the general relay from enzyme supply during koji to salt-phase metabolism during moromi [82].

### 3.5. Douchi-Qu Communities, Typified Assembly

Douchi fermentation followed three stages with an initial starter, a natural microbial pool, and salt-driven post-ripening. Community assembly showed distinct typification. By dominant microbes, it was grouped as *Aspergillus*-type, *Mucor* or *Rhizopus*-type, and bacterial-type, with some sources listing non-inoculated and bacteria-led products as the bacterial type. This classification matched the types of starters and environmental exposure and determined dominant phyla and metabolic potential downstream. Recent work found that industrial and household samples contained all three lineages, with *Aspergillus*-type and bacterial-type being the most frequent. These two showed different emphases in aroma and amino-acid-release routes. In two traditional bacterial-type douchi from Gansu, 16S and ITS data showed *Bacillus*, *Ignatzschineria*, *Proteus*, and *Providencia* as dominant bacteria. *Pichia*, *Candida*, and *Rhodosporidium* were dominant fungi. This indicated that without a mold starter, bacteria and yeasts could still build a backbone network for fermentation and flavor [89].

In *Aspergillus*-type douchi, community succession showed a reproducible turn from molds to yeasts or bacteria. High-throughput tracking of Yangjiang samples showed that molds accounted for about 70% of the fungi during early koji making. Yeasts then rose rapidly in later fermentation and reached about 90%. The two then approached balance at the end. This indicated niche replacement within fungi and staged differences in substrate use [90]. At finer time scales, the koji stage was dominated by Staphylococcus and *Weissella*. Later, *Bacillus*, *Corynebacterium*, and *Acinetobacter* rose. *Weissella* rose from 34.05% to 48.79% on days 1–3 then fell to 2.6% and rose again to 15%. This reflected short-cycle fluctuations under salt penetration and substrate shifts. At the phylum level, bacteria were dominated by Firmicutes, Actinobacteria, and Proteobacteria. Fungi were dominated by Ascomycota and Zygomycota [91,92,93]. These results indicated that the starter not only supplied early enzymes but also built a micro-ecological base through a mold-dominant window for later salt- and acid-tolerant members to take over. Compared with *Aspergillus*-type, *Mucor* or *Rhizopus*-type samples showed different dominant mold sets and co-occurrence networks during the koji stage. A comparison of the two types reported that *Aspergillus*, *Candida*, Meyerozyma, and Lecanicillium shared dominant fungal genera in more than half the samples during koji. Relative abundance and interaction strength differed systematically between types. Although that work focused on taste indices, its community-level results provided direct evidence for typified assembly [94]. Bacterial-type douchi was seen as a no-mold or bacteria-led model. Across regions, Firmicutes dominated. *Bacillus* was often the dominant genus. *Candida*, *Millerozyma*, and *Lichtheimia* co-appeared in some samples, indicating a bacteria-led sample with yeast-supported symbiosis [95,96]. In summary, *Aspergillus*-type douchi formed a stepwise replacement of molds, yeasts, and bacteria during making and fermentation, *Mucor* or *Rhizopus*-type differed in the mold set during koji with resulting differences in taste precursors, and bacterial-type used *Bacillus* and related Gram-positive rods as the backbone with osmotolerant yeasts to deepen flavor during post-ripening.

### 3.6. Sufu Pehtze Communities, Mold–Bacterium Synergy for Texture and Flavor

Sufu production started from molded tofu, or pehtze. It was a filamentous fungus-led system that formed early interactions with bacteria. Sequencing across stages showed that the pehtze fungus community was built around the inoculated mold and held absolute dominance. In mixed black- and yellow-soy sufu, *Rhizopus* reached 77.96% and 93.35% relative abundance at the pehtze and salted-pehtze nodes. At the phylum level, *Mucoromycota* was enriched during pre-fermentation, while Ascomycota and Basidiomycota were enriched during later stages. Bacteria kept high alpha diversity throughout. This indicated that while molds controlled structure during pehtze, a bacterial baseline already existed and influenced later flavor and texture. In the mid to late post-fermentation, bacterial communities converged toward Enterococcus, Enterobacter, and in some cases *Bacillus*. This showed a path of early mold dominance followed by bacterial convergence [97].

From a community–function view, extracellular hydrolases formed during pehtze set the substrate supply for later softening and flavor precursors and shaped niches for companion bacteria. In full-factor tests at 25–35 °C and 73–97% RH over 12–60 h, *Rhizopus* oligosporus reached growth and enzyme activities comparable to *Actinomucor* elegans at temperatures of about 10 °C higher. This suggested ecological substitution potential in hot seasons. These quantitative results across temperature, humidity, time, and enzyme output explained why *Actinomucor* commonly laid the fine basis for cheese-like texture and why introducing *Rhizopus* under hot conditions still maintained protein and oligosaccharide cleavage and supplied amino acids and fermentable sugars for later bacterial metabolism [98].

Bacteria were not absent during pehtze. They acted as molded collaborators. Classic surveys of commercial sufu showed LAB at 10^5^–10^7^ CFU/g in white sufu, indicating that salt- and acid-tolerant bacteria gradually took over parts of acidification, amino acid decarboxylation or conversion, and ester-precursor supply across pehtze, salting, and ripening. In line with this, community comparisons of commercial red and white sufu found significant differences in relative abundance of Firmicutes and Proteobacteria at *p* < 0.01 and significant genus-level differences for *Lactococcus* and Tetragenococcus between products at *p* < 0.01. This revealed directions of bacterial convergence under high salt and suggested that amino acid and organic-acid spectra formed by molds during pehtze exerted feed-forward effects on bacterial assembly in different product lines [99,100].

Core pehtze fungi had been delineated for sources and key members. Identification of commercial starter molds from factories in provinces of China and in Vietnam showed *Actinomucor repens*, *A. taiwanensis*, *Mucor circinelloides*, *M. hiemalis*, *M. racemosus*, and *Rhizopus microsporus* var. microsporus as a stable starter pool. Phylogeny showed *Mucor* and *Actinomucor* as closer and *Rhizopus* as more distant. The cross-regional stability of this pool explained the commonality of similar appearance and texture at the pehtze stage, while later bacterial differences by region appeared more in salting and ripening stages through metabolic and flavor divergence [101].

Overall, the sufu pehtze ecology followed three elements. Molds laid the base. Core filamentous fungi such as *Actinomucor*, *Mucor*, and *Rhizopus* released high-throughput proteases and glycosidases within 24–60 h and supplied utilizable nitrogen and oligosaccharides. Bacteria were pre-loaded. They remained at low abundance and high diversity early, then converged under high-salt and low-oxygen selection toward LAB and *Bacillus* and took over. Co-formation of flavor and texture followed. Enzyme flux and substrate spectra during pehtze set the material landscape for later acidification and esterification and drove the depth of cheese-like texture and flavor. This self-organized mold–bacterium synergy was indicated by stage-wise relative abundance values such as *Rhizopus* at 77.96–93.35% [97].

**Figure 2:**
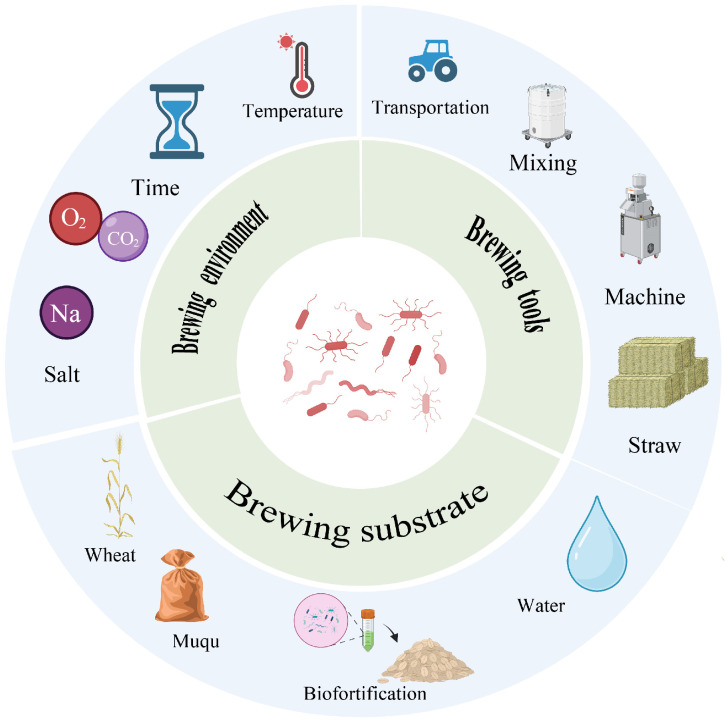
Linkage between engineering factors and microbial functions.

As shown in Table 1 and Figure 2, the community structures of the various starter cultures were shaped by environmental filtering dominated by heat history, substrate, and salinity, and were coupled to flavor formation and quality stability through a spatiotemporal relay of enzyme production and conversion. In summary, the temperature type of daqu acted as the primary constraint on assembly and functional differentiation. High-temperature daqu enriched Firmicutes and thermotolerant fungi under heat pulses and drove protein and polysaccharide cleavage and pyrazine-related metabolism, whereas low-temperature daqu favored a mild platform of yeasts and lactic acid bacteria, reflecting an ecology guided by thermal history. Xiaoqu formed a compact triad composed of *Rhizopus*, *Saccharomyces*, and lactic acid bacteria that rapidly completed the relay from saccharification to ethanol production and acidification. Hongqu was centered on *Monascus*, which regulated pigment and polyketide networks through secondary-metabolism gene clusters and interacted with yeasts over time. The soy sauce system exhibited a two-stage relay in which the koji stage supplied enzymes and the moromi stage selected salt-tolerant metabolism, with Tetragenococcus and *Zygosaccharomyces* gaining ecological advantage under high salt to drive acidification and the formation of key flavor precursors. Douchi and sufu showed, respectively, typified assembly and a cooperative path in which molds laid the foundation and bacteria followed, thereby completing a continuous ecological design from the initial starter to late maturation.

## 4. Food Application Spectrum Centered on Qu

### 4.1. Daqu as a General Engine for Distilled Spirits and Grain Vinegars

#### 4.1.1. Coupling Between Baijiu Aroma Style and the Daqu Temperature Regime

**Figure 3:**
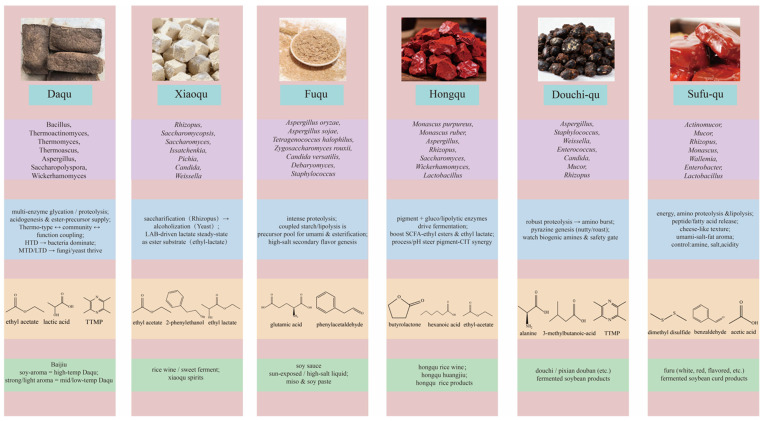
Schematic of the food application spectrum centered on qu.

As shown in Figure 3, in sauce-aroma baijiu production, high-temperature daqu with peak making temperatures of 60–65 °C provided thermotolerant enzyme systems and, through piling and airing before cellaring, promoted Maillard reactions and pyrazine formation. This formed the base notes of sauce, roasted, and toasted characters. The process used stronger daqu dosing. A daqu to raw material ratio near 1:1 was reported as an industry trait. This helped maintain high enzyme activity and precursor supply across multiple fermentation rounds.

During piling, the heap temperature rose to 48–55 °C before cellaring. Each round in-cellar lasted about 30 days. The full cycle often reached seven rounds. This reflected high daqu use, high temperature, and many rounds. The link between high-temperature daqu and directed pyrazine formation was quantified. By contrast, strong-aroma practice relied on medium-temperature daqu. The pit–mash system targeted high ethyl acetate and ethyl hexanoate. Recent work optimized parameters from the perspective of high ethyl hexanoate production and verified coupling with daqu dose and piling strategy. Medium-temperature daqu often acted with functional strains in solid-state systems; for example, yeasts selected for high ethyl hexanoate. Light-aroma practice relied more on the saccharification–alcohol fermentation efficiency of low- or medium-temperature daqu and on clean flavor output. Dependence on long pit aging was lower. The process emphasized stable saccharifying power and neutral or acidic protease activity of the qu [102]. Notably, different “color qu” inside high-temperature daqu, namely white, yellow, and black, showed measurable differences in saccharifying enzymes and neutral proteases. White qu was higher. This suggested that blending by inner-type and ratio allowed fine-tuning of flavor and buffering of quality variation during base spirit design [103,104,105,106,107,108,109,110,111]. These parameterized data connected temperature regime, daqu dose, heap temperature, number of rounds, and key flavor compounds to executable operating windows.

#### 4.1.2. Daqu in Traditional Grain Vinegars

In grain vinegar exemplified by Shanxi aged vinegar and Zhenjiang aromatic vinegar, daqu based on barley or pea, also called vinegar qu or wheat qu, first drove starch saccharification and alcohol formation, and then the process entered solid-state acetification. In the Shanxi aged vinegar specification, total acid stayed at or above 6 g/100 mL over years. This reflected efficient docking across qu, alcohol, and vinegar stages. In comparison with Zhenjiang aromatic vinegar, a dry-gelatinization route reached 16.2% *v*/*v* ethanol within five days versus six days and 13.9% by a traditional route. During acetification, total acid reached 7.178% *w*/*v* and 7.092% *w*/*v* at 18–20 days. This showed that higher upstream saccharification and alcohol conversion translated to stronger acid formation later. Other work found total acid commonly at 3.5–8.0 g/100 mL in grain vinegars. Acetic and lactic acids usually exceed 80% of total organic acids. Their ratio tracked the saccharified products such as glucose and dextrins and the ethanol formation rate during alcohol fermentation. Industry studies on community–process coupling in Zhenjiang aromatic vinegar across seasons and with qu addition or inoculation strategies confirmed that daqu quality and dosing altered the initial alcohol level and the slope of acid rise. They also changed the accumulation rates of key precursors such as acetoin and diacetyl. This set the final landscape of free amino acids and organic acids [112,113,114]. In short, the three-stage chain centered on qu in grain vinegar could be tuned directly and quantitatively to target acid and flavor by choosing the qu type, dosing, pretreatment, and inoculation strategy.

### 4.2. Xiaoqu in Southern Rice-Based Beverages and Local Baijiu

#### 4.2.1. Household and Workshop Systems Such as Rice Wine, Sweet Rice Wine, and Black Glutinous Rice Wine

In rice-based beverages centered on xiaoqu, process control did not target high alcohol. It targeted a balance of high sugars, low alcohol, and soft acidity. This matched high saccharifying activity, mild ethanol formation, and moderate acidification supplied by xiaoqu. In sweet rice wine, recent evaluations showed low alcohol across treatments when traditional sweet xiaoqu was used. All groups stayed below 2.0% vol. This matched low conversion by xiaoqu yeasts and a preference to retain sugars, which suited sweet and glutinous products used in desserts or warm drinks [115]. Time–temperature windows were compressed into short dual-fermentation periods of 24–48 h. A representative study tracked 0, 24, 36, and 43 h and observed a steady fall of soluble sugars with rising total acid and slow alcohol accumulation. This verified the cooperative path of *Rhizopus*-led saccharification, *Saccharomyces* alcohol formation, and LAB mild acidification. The design met household or workshop needs for fast turnaround and reduced contamination risks from long cycles [116]. Formulation and substrate adjustments also worked. Adding pretreated rice bran by extrusion to sweet rice wine increased soluble solids and antioxidant indices without changing the low-alcohol trait, which stayed below 2% vol. This provided a handle to enrich flavor and nutrition for low-strength sweet profiles [115].

On the supply side of xiaoqu function, key hydrolases from *Rhizopus* in traditional rice wine starters differed by orders of magnitude among regions. This set sugar release rates and controllability of final sweetness in short fermentations. In a survey of 29 regions, 78 *Rhizopus arrhizus* isolates from rice wine qu showed acidic protease at 280–1023 U/g, amylase at 557–1681 U/g, and esterase at 370–2949 U/g. The wide span indicated that selecting qu and process in combination could set saccharification strength and esterification potential with precision. These data supported style settings from home or workshop to small industry [117]. In black glutinous rice wine, xiaoqu from Sichuan, Jiangxi, and Hubei used on the same substrate yielded 61 volatile compounds in total, with 16 at OAV > 0.1. Functional differences among regional xiaoqu shifted the relative contributions of ethyl acetate, ethyl lactate, and 2-phenylethanol. This produced clear sensory divergence under the same recipe and different qu, which supported regional product development and mapping of standardized flavor spaces [118]. In sum, the engineering value of xiaoqu in rice beverages lay in stable output of sweet, low-alcohol, and clean aromas through three-way coupling of short temperature control often near 28–30 °C, adjustable enzyme activity by qu selection, and material pretreatment by steaming, extrusion, or gelatinization. Quantified enzyme and schedule parameters supported scale-up [42,115].

#### 4.2.2. Xiaoqu Baijiu and Regional Styles

Solid-state making of xiaoqu baijiu, represented by rice-aroma baijiu, followed xiaoqu use, semi-solid fermentation, and solid distillation. Key flavor groups including higher alcohols and esters such as ethyl acetate and ethyl lactate were highly sensitive to the source and properties of xiaoqu. In a production description for a rice-aroma line, xiaoqu supplied starch hydrolysis and primary precursors early. A semi-solid fermentation of about 13 days completed alcohol formation before distillation. The coupling between temperature and total acid acted as the main external drivers for shifts in communities and metabolic flux. In plant samples, *Lactobacillus* reached 62.88–99.23% at the end of fermentation. *Saccharomyces* and *Rhizopus* on the fungal side ranged 7.06–83.50% and 15.21–90.89%. These showed significant positive associations with ethyl acetate, ethyl lactate, and total acid in redundancy analyses, which supported engineering control of flavor by process variables [76]. Choice of xiaoqu for style targets also pulled a higher alcohol to ester balance. A comparison of six commercial xiaoqu showed ethyl acetate and ethyl lactate rising rapidly early, leveling later, and jumping after distillation. Redundancy analysis indicated strong association between *Saccharomyces* from qu and the formation of these two esters and higher alcohols. This showed a direct effect of qu diversity on final distributions via the qu–yeast–ester synthase path and guided selection for routes that targeted low higher alcohols and high esters [119].

Quantitative flavor profiles from cross-category and within-style statistics placed xiaoqu systems at high ester and moderate higher alcohol levels. A systematic evaluation of rice-aroma baijiu reported a typical ratio of total acid to total ester to total alcohol near 1:1.2:1.5. Lactic and acetic acids exceeded 90% of total acids. Esters were led by ethyl lactate, followed by ethyl acetate. 2-Phenylethanol was among the highest of the four main baijiu styles. These ratios guided cut points during distillation and blending [2,120]. Raw materials and process also pulled targets. In black rice wine, regional xiaoqu with the same substrate delivered reproducible differences in 16 OAV > 0.1 markers by intensity and profile. Moving this qu effect into rice-aroma baijiu pilots allowed a higher ethyl acetate to ethyl lactate ratio to improve freshness and top-note lift [118,121]. Combining qu addition, mash moisture, and time was used to constrain total higher alcohols into a sensory-friendly zone. Composite qu and redesigned fermentation windows kept total higher alcohols at several hundred mg/L and raised ethyl esters. This achieved refined and elegant regional expressions such as Lingnan rice-aroma without sacrificing body [119,122]. In general, regional style in xiaoqu baijiu depended not only on materials and distillation or blending but also on engineered coupling of qu diversity, process variables, and distillation cuts. Parameter windows centered on the ratio of ethyl acetate to ethyl lactate and thresholds for higher alcohols used qu choice, temperature and acid control, and water management as core levers.

### 4.3. Hongqu, a Combined Platform for Pigment and Aroma

#### 4.3.1. Hongqu Rice Wine and Hongqu Huangjiu

In hongqu rice wine and hongqu huangjiu, hongqu performed saccharification and pigment formation and linked positively with ester generation. In a recent pilot model that used pure cultures together with hongqu, co-fermentation by *Monascus purpureus* and *Saccharomyces cerevisiae* yielded 29 volatiles. Esters dominated with ten species, and ethyl acetate was the leading ester. With 25% Trichosanthes fruit pulp added as a flavor and enzymatic co-substrate, ethyl acetate still dominated. Ethyl lactate, methyl heptanoate, and lactones appeared. Ethyl lactate made up 12% of total esters. For higher alcohols, 3-methyl-1-butanol and 2-phenylethanol reached 36.08 μg/mL and 8.71 μg/mL by SPME–GC–MS at room sampling. This showed that under parallel saccharification and alcohol fermentation with hongqu, esters and alcohols could be raised by formulation without sacrificing cleanliness or color stability. The work also summarized 81 and 102 total volatiles in traditional reports, suggesting that mixed-culture fermentations had richer spaces than pure cultures. This matched process reliance on qu complexity. These data provided anchors for co-optimizing process strengthening such as co-fermenting materials or adding flavor substrates and flavor targets such as high ester ratios [123].

Beyond flavor, pigment production and enzyme flux could be tuned quantitatively by formulation and process. Response surface optimization for *M. purpureus* G11 in liquid culture on a mix of rice flour, peptone, copper ions, and vitamin B1 gave a theoretical pigment yield of 113.77 U/mL and a saccharification-related SP activity of 362.91 U/mL. This supported industrial stability of color and precursors by linking functional seed culture and qu rooms. Although obtained in liquid, the directions of effects across carbon, nitrogen, trace elements, and cofactors informed solid-state scale-up and guided pretreatment and moisture management of qu bricks [124].

#### 4.3.2. Hongqu Vinegar and Hongqu Sufu

In hongqu vinegar, hongqu first completed saccharification and alcohol formation; then, the mash entered acetification. Metabolic studies of traditional practice resolved 60 volatiles, 23 amino acids, and seven organic acids across the process. Acetic acid, lactic acid, and esters of these acids accumulated in the middle and late stages and set total acid and aroma structure. This three-stage interface gave process rationale for using hongqu on the raw material side [125].

In sufu, hongqu rice and pigments raised color directly and altered organic acid spectra and flavor composition. A multi-site survey of commercial red sufu and white sufu showed lactic acid as the main acid. Lactic acid was 1.969 ± 0.300 g/kg in red sufu and 2.790 ± 0.612 g/kg in white sufu. Acetic acid was 0.715 ± 0.102 g/kg and 0.855 ± 0.090 g/kg. Citric acid was 0.805 ± 0.050 g/kg and 0.679 ± 0.153 g/kg. This suggested that hongqu contributed not only color but also shifts in organic acids and volatiles via substrate and osmotic effects, which shaped final flavor [99].

### 4.4. Fuqu or Jiangqu as the Salt-Tolerant Core for Soy Sauce and Soybean Pastes

#### 4.4.1. Soy Sauce Koji with *Aspergillus oryzae* or *A. sojae*

In the soy sauce process, mature koji was combined with brine to form moromi. Salinity fell in the high-salt liquid fermentation zone to ensure safety and allow long maturation. Salt levels of about 18–23% *w*/*v* NaCl were reported. Maturation ranged from months to at least four years according to process and product positioning. Neutral and acidic proteases, glutaminase, α-amylase, and glycosidases produced during koji still supplied substrates under high salt and low water activity. This drives amino nitrogen and key flavor-threshold compounds during aging. Under the JAS grading for soy sauce nitrogen at the linked site, grades centered on total nitrogen and amino nitrogen. This supported the industrial practice of securing nitrogen indices by ensuring enzyme output during koji. Process trials showed that moromi temperature and intermittent aeration changed alcohol and acid metabolism. In a comparison between 25, 35, and 45 °C, the higher temperature at 45 °C accelerated changes in aging indices but lowered ethanol and did not change total nitrogen significantly. This suggested that temperature and aeration should be balanced to maintain flavor and stability in high salt. Traditional sun fermentation used outdoor insolation and stirring to promote mass transfer and redox reactions. Modern work still treated natural sun fermentation as a basis for traditional premium flavor [53,126,127,128]. These windows of temperature, salt, and time gave an operable interface from koji to moromi. Producers therefore optimized enzyme output upstream with the end in mind to meet amino nitrogen and characteristic aroma grades later.

#### 4.4.2. Doubanjiang, Huangdoujiang, and Tianmianjiang

In high-salt solid or semi-solid pastes, fuqu served as an enzyme engine that set nitrogen release and flavor potential. The Codex regional standard set floors for amino nitrogen and total nitrogen in fermented soybean pastes. For pure soybean pastes, amino nitrogen had to be at least 0.30% *w*/*w* and total nitrogen at least 1.6% *w*/*w*. For soybean plus cereals, amino nitrogen had to be at least 0.12% *w*/*w*. These indices correlated with proteolysis intensity during koji. Producers therefore raised effective neutral and acidic proteases by managing moisture, layer thickness, and turning to improve amino nitrogen and stabilize free amino acid release [129].

In industrial Sichuan-style douban, a closed gradient steady thermal field over 90 days detected 75 volatiles, compared with 67 in constant temperature and 68 in traditional outdoor processes. The closed system showed more esters and alcohols. This suggested that engineered gradients and ventilative stirring improved accessibility and mass transfer for ester precursors and thus improved layering of paste and ester notes. Work on salt reduction or replacement showed that under controlled risk and stable operation, low-salt options could somewhat raise amino nitrogen or adjust volatiles but required stronger enzyme output and tighter monitoring during koji. Mechanistic work also showed continued peptide degradation and amino acid accumulation during the high-osmotic moromi stage of douban. This indicated a relay of extracellular proteases from koji into the salt-tolerant stage and formed the key link from qu to amino nitrogen to volatiles [129].

### 4.5. Douchi-Qu, Community Differences, and Quality Indicators in Aspergillus-Type, Mucor or Rhizopus-Type, and Bacterial-Type Douchi

In industrial contexts, the qu type set community assembly and the output profile of flavor in douchi. Horizontal comparisons of commercial products found both shared volatile foundations and type-specific fingerprints. In a comparison across mold-type douchi, the three types shared 26 volatiles. Unique compounds also appeared, with nine in *Aspergillus*-type, seven in *Mucor*-type, and nine in *Rhizopus*-type. *Aspergillus*-type had higher free amino acids and umami notes with *p* < 0.01. This suggested a process-amplifiable coupling between qu, community, and flavor [130].

For *Aspergillus*-type, the route included making the starter or bricks, post-ripening, and drying. Community succession and aroma formation showed stage windows. Tracking through the process found molds near 70% in early making. Yeasts rose later and approached 90%. The two then balanced near the end. Across five steps, 83 volatiles were identified, including 20 alcohols, 12 aldehydes, eight ketones, 15 esters, and eight aromatics. The profile shifted from bean-derived alcohols and aldehydes such as 1-hexanol, 1-octen-3-ol, and nonanal to phenolics and ketones that raised paste, smoky, and nutty notes. This mold-to-yeast relay gave clear handles for control [90].

Mucor or *Rhizopus*-type emphasized a composite base of yogurt-like acid, caramel, and smoke. In Yongchuan *Mucor*-type douchi, GC–O, AEDA, and OAV identified 49 odor-active compounds, with 22 at high FD 8–2048. Acetic acid at 535.58 μg/g and maltol at 153.23 μg/g led by amount. Dimethyl trisulfide at OAV 8818 and 2-methoxyphenol at OAV 1317 contributed strongly to aroma. This combination of organic acids, sulfur compounds, and phenolics set the key dimensions that separated *Mucor*-type from *Aspergillus*-type by e-nose and sensory [131]. For *Rhizopus*-type, evidence showed shifts toward caramelized ketones, sesquiterpenes, and some pyrazines. In a comparison of pure and mixed cultures, 5% *Rhizopus arrhizus* at 30 °C in solid state for eight days formed different key volatiles such as 2,3-butanedione, dihydro-3-methyl-2(3H)-furanone, and α-pinene. Co-fermentation with *Aspergillus* or *Bacillus* broadened ester and ketone coverage. This suggested potential to expand aroma by strain–process synergy in short-cycle and strong-modification settings [132].

Bacterial-type led by *Bacillus* showed industrial readability in communities and safety–quality indices. In “water douchi” under room temperature natural fermentation, total bacteria averaged 1.6 × 10^6^ CFU/g, LAB 5.9 × 10^4^ CFU/g, and fungi 7.6 × 10^3^ CFU/g. Amino nitrogen reached 2.08 g/kg, titratable acid 3.44 g/kg, and reducing sugars 25.78 g/kg. Polyamines were detected such as putrescine at 131.3 mg/kg, cadaverine at 83.4 mg/kg, and tryptamine at 61.5 mg/kg. These gave thresholds and alerts for raw materials and salt–temperature–aeration strategies. Two traditional bacterial-type douchi from Longnan and Qingyang differed. Forty-eight and forty-one volatiles were detected. ROAV-selected key odorants formed different sets by region. *Carnobacterium*, *Ignatzschineria*, and *Bacillus* dominated. This supported the view that bacterial-type formed a savory and salty base with ester and acid extension [89].

In summary, *Aspergillus*-type suited staged temperature and humidity control to yield a rich profile with yeast-led post-ripening and amplified phenolics and ketones. *Mucor* or *Rhizopus*-type anchored yogurt-like acid, smoke, and caramel clusters and suited short cycles or seasonal windows to raise key OAV compounds. Bacterial-type featured high amino nitrogen and clear microbial and physicochemical thresholds that enabled data-driven control and safety evaluation. Industrial choice among the three should integrate target flavor indices such as OAV > 10 sulfur or phenolics and ester or pyrazine coverage, community stability, and quality and safety indices including amino nitrogen, acidity, and amines.

### 4.6. Sufu-Qu, the Cheese-like Path of Molded Tofu

In modern sufu production, sufu-qu pre-digested tofu blocks during a short solid-state stage. This supplied soluble nitrogen, fermentable and esterifiable substrates, and texture softening for long maturation in salty alcoholic brine. For hot seasons, different rooms, and flavor targets, the pehtze stage used division of labor among qu sources to control enzyme windows. With *Actinomucor* elegans, peak protease of 108 U/g, lipase of 172 U/g, and glutaminase of 176 U/g were reached at 25 °C, 95–97% RH, and 48 h. To raise saccharification and oligosaccharide removal, α-amylase of 279 U/g and α-galactosidase of 227 U/g were reached at 30 °C and 95–97% RH at 48 h and 60 h. When ambient temperature rose to 30–35 °C in summer, *Rhizopus oligosporus* served as a thermotolerant alternative. At 35 °C and 95–97% RH, peak protease and lipase reached 104 U/g and 187 U/g at 48 h. At 36 h, α-amylase and glutaminase reached 288 U/g and 187 U/g. α-Galactosidase reached 226 U/g at 30 °C and 36 h. These quantified enzyme–time–temperature–humidity coordinates supported room settings and seasonal switching and linked enzyme output at the qu stage to later flavor formation [98].

After pehtze, maturation proceeded in brine with salt, alcohol, and spices. Extracellular and some intracellular enzymes from pehtze continued to act under high salt and low water activity. Emulsification, protein breakdown, and small flavor precursors proceeded. In 23 commercial sufu, NaCl ranged 6.2–14.8% *w*/*w* and ethanol 0.5–6.3% *w*/*w*. Free amino nitrogen and reducing sugars such as glucose at 0–6.2% and fructose at 0–4.8% varied. These endpoint ranges across brands confirmed the need to convert insoluble macromolecules into soluble nitrogen and esterification substrates during pehtze. They also set benchmarks for maturity criteria before brining, such as lower moisture and elasticity and higher soluble nitrogen [100].

In the process chain, sufu-qu also standardized interfaces between product categories and styles. Commercial practice cut tofu is cultivated for two to seven days at 12–30 °C to form pehtze covered evenly by hyphae. Maturation then occurred in salty alcoholic brine for months. Starter identification across *Actinomucor*, *Mucor*, and *Rhizopus* showed industrial strains of *Actinomucor repens* or *A. taiwanensis*, *Mucor circinelloides* or *M. hiemalis* or *M. racemosus*, and *Rhizopus microsporus*. The clades were clear and matched process tolerance and enzyme spectra. This chain of cultivation schedules, brine maturation, and starter phylogeny supported reproducible standards for raw material acceptance, qu selection, and environmental tolerance at the pehtze stage [101].

Viewed from the product end, the levels of volatiles and soluble nitrogen after maturation were largely set by enzyme flux and structural remodeling during pehtze. In a model with black and yellow soybeans at 1:1, after 60 days in brine, 79 volatiles were resolved including 31 esters and nine alcohols. Seventeen free amino acids were detected. Coupled SPME–GC/MS and sequencing proved that protein and lipid cleavage during pehtze provided the base for later ester formation and umami amino acids [97]. Producers could therefore scale the three segments of pehtze parameters, brine formulation, and aging time to quantitative targets for ester fractions and amino nitrogen thresholds.

In sum, sufu-qu process engineering was summarized as maximizing target enzyme systems and texture modification in a short solid-state period and transferring that potential smoothly into long maturation in high-salt alcoholic brine. Producers could switch seasonally between *Actinomucor* and *Rhizopus* according to ambient temperature and flavor targets, lock in 25, 30, or 35 °C at 95–97% RH for 36–60 h as optimal enzyme windows, and back-calculate pehtze maturity and brining timing from endpoint ranges such as 6.2–14.8% NaCl and 0.5–6.3% ethanol. This achieved stable cheese-like texture and layered flavor output.

## 5. Cross-Scale Coupling Between Functional Enzyme Spectra and Flavor Formation

### 5.1. A Synergistic Network of Saccharification, Alcohol Fermentation, Proteolysis, and Lipolysis with Flavor Routes

Flavor in Chinese qu-based fermentations was produced by a synergistic enzyme network. In solid-state systems, starches, proteins, and lipids were hydrolyzed to small molecules by microbial enzymes. These intermediates were converted through central metabolism and released complex flavor compounds. Carbohydrates were first saccharified to provide sugars and energy. Amylases hydrolyzed starch to fermentable sugars. Yeasts converted sugars through glycolysis and the TCA cycle to pyruvate and ethanol. Acetyl CoA served as a key precursor for ester synthesis. In the presence of yeast ethanol, esterases generated ethyl acetate and related aromatic esters. In strong-aroma baijiu, ethyl hexanoate formed from hexanoic acid and ethanol through enzymatic esterification [133,134]. During fermentation, saccharification and alcohol formation proceeded in parallel. Molds supplied sugars and yeasts produced alcohol. Their products entered subsequent reactions. LAB converted glucose to lactic acid. Lactic acid then esterified with yeast ethanol to form ethyl lactate and increased body. Measurements showed that fortified qu enhanced cooperative ester formation by LAB and yeasts, and one trial group reached 128.01 mg/L ethyl lactate [135,136,137].

Proteins were hydrolyzed by proteases to amino acids and peptides. These provided nitrogen for yeasts and LAB and also acted as taste components and volatile precursors. Glutamate contributed umami flavors. Branched chain amino acids entered the Ehrlich route and yielded higher alcohols such as isoamyl alcohol. Protease activity shaped the types and levels of alcohols and organic acids and thus the amounts and ratios of esters, which shifted flavor trajectories. In high-temperature qu, abundant protein breakdown products reacted with sugars under suitable heat and moisture through Maillard chemistry to produce nitrogen heterocycles such as pyrazines and gave nutty roasted notes. Tetramethylpyrazine was a typical Maillard product that imparted roasted and nutty characters in sauce-aroma qu. Lipids were hydrolyzed by lipases to glycerol and free fatty acids. These lipolysis products served as energy sources and also contributed flavor. Short-chain fatty acids such as acetic, butyric, and hexanoic acids were important to qu aroma. Hexanoic acid carried sharp acidic notes. Free fatty acids esterified with alcohols under esterases to raise aromatic intensity with fruity and floral notes. Adding exogenous lipase to the system significantly promoted esterification and increased flavor ester concentrations in baijiu by up to thirty-one-fold.

Microbial roles were divided in traditional qu mashes. Bacilli with strong enzyme production secreted amylases, proteases, and lipases to hydrolyze substrates and supplied sugars, amino acids, and fatty acids as precursors. They also produced acids and aroma directly. *Bacillus* licheniformis in solid-state fermentation generated aromatic compounds, C4 short-chain compounds, pyrazines, and volatile organic acids. *Aspergillus* strains in qu produced broad spectrum hydrolases, saccharified starch, hydrolyzed proteins, and transformed polyphenols. When *Bacillus* and *Aspergillus* co-fermented, complementary enzymes accelerated aroma metabolism and increased phenols, aldehydes, and other complex volatiles. Cascades also linked metabolites together. LAB produced lactic acid that yeasts esterified to ethyl lactate and softened body. Balanced LAB with hexanoic acid producers favored hexanoate accumulation, which then combined with ethanol to form ethyl hexanoate with a clean and rich bouquet.

In sum, saccharification, alcohol formation, proteolysis, and lipolysis in qu formed an interwoven network. Degradation products served as substrates for the next group of microbes and were converted stepwise to higher alcohols such as 1 butanol and isoamyl alcohol with green apple or fruity hints, to organic acids such as acetic and butyric acids that gave acidity and irritation when excessive, and to esters such as ethyl acetate and ethyl hexanoate that provided floral, honeyed, and fruity notes. Minor nitrogen volatiles such as ammonia, pyrazines, and indole contributed distinctive fermented aromas and built layered sensory quality.

### 5.2. Enzyme–Substrate–Microbe Interactions from Substrate Specificity to Molecular Origins of Aroma and Taste

Substrate composition selected colonizing microbes and enzyme expression and set molecular starting points and routes for flavor. In starch-led systems such as grain-based daqu or hongqu, starch-rich substrates favored amylase-producing molds such as *Aspergillus* and *Rhizopus*. Rapid saccharification supplied sugars. Osmotolerant yeasts such as *Saccharomyces* and film-forming yeasts grew well and converted sugars to ethanol and organic acids. Enzymes then transformed these intermediates to diverse volatiles. This substrate bias produced aroma spectra dominated by alcohols and esters. High ethanol and various organic acids formed ethyl acetate, ethyl lactate, ethyl hexanoate, and related esters under esterases and gave rich fruity and full-bodied profiles. In medium-temperature daqu made from starchy materials, 328 volatile compounds were detected, whereas only 140 were detected in low-temperature qu. This showed that starch-rich environments with suitable microbial synergy produced richer flavor components [138,139,140,141]. Hongqu rice wine used glutinous rice and followed the same starch-led logic. Its profile featured mellow, smooth, and sweet traits that matched alcohols and esters formed from fully fermented saccharification products [142]. Traditional hongqu huangjiu contained more than one hundred volatiles, with reports up to 102 compounds that included higher alcohols such as isoamyl alcohol and 2 phenylethanol, esters such as ethyl acetate and ethyl phenylacetate, and minor aldehydes and ketones. These built the characteristic aroma and lingering sweetness of hongqu wines [143].

Protein- and lipid-led fermentations such as sufu followed different flavor routes. Starch in fermented tofu was minimal. Molds expressed proteases and lipases rather than amylases. Filamentous fungi such as *Mucor* and *Aspergillus* grew on tofu and secreted proteases with high activity. *Mucor* proteases exceeded those in other fermentations and hydrolyzed soybean proteins to small peptides and amino acids. Strains also produced lipases that released abundant fatty acids [144]. Under these substrates, microbes including later brine bacteria used amino acids and fatty acids for metabolism and generated nitrogen- and oxygen-containing flavor compounds. Proteases from molds and bacteria released amino acids that supplied sour, sweet, bitter, salty, and umami tastes and acted as precursors. Through deamination, transamination, and chain cleavage, different amino acids yielded aldehydes, ketones, alcohols, and organic acids. Together with lipid products, they formed the composite aroma of sufu. Accumulation of alanine and other amino acids brought slight sweetness. Long maturation increased ester formation and added fruity notes. In deeply fermented products such as stinky tofu, this appeared as a sweet and mellow trait. Roles were divided among microbes. Leuconostoc like cocci and members of Clostridiaceae contributed short-chain acids and esters. Enterobacteriaceae and Enterococcus promoted flavor amino acids and reducing sugars. LAB and molds produced and accumulated lipases that catalyzed lipid hydrolysis and released fatty acids. During ripening, these fatty acids esterified with alcohols present in brine and produced esters such as ethyl hexanoate that gave buttery richness. Salt and huangjiu added to the brine also affected enzyme–substrate action. High salt suppressed microbial activity and reduced amino acid formation, which lowered alcohols, ketones, and phenolics. Moderate addition of rice wine or distilled spirits introduced yeasts for minor alcohol fermentation and supplied alcohol for esterification to raise aroma complexity.

Thus, substrate composition drove microbial ecology and enzyme expression. Starch-rich qu favored saccharification and alcohol routes and produced vinous and fruity notes. Protein- and lipid-rich qu favored proteolysis and lipolysis and produced umami notes from amino acids, special odors from ammonia and amines, and buttery notes from lipid oxidation products. Multi-omics work confirmed that functional communities in different qu adjusted enzyme spectra to substrates. Integrated meta-omics and transcriptomics revealed substrate-specific expression of key enzymes and activation of metabolic pathways. Yi et al. [24] reported up to 11 aromatic compound pathways activated in HT-Daqu to break down and use complex aromatic precursors. Kang et al. [145] resolved assembly mechanisms in jiuqu communities and clarified functional partitioning and enzymatic cooperation among populations under different raw materials. In sum, enzyme–substrate–microbe interactions set the molecular origins of flavor. Substrate types and ratios shaped microbial ecology and functional enzymes. These governed formation and conversion of flavor precursors. Elucidating this cross-scale linkage supported targeted control of qu enzyme composition and fermentation strategies to optimize flavor quality in traditional fermented foods.

## 6. Quality and Safety from Risk Identification to Process Control

### 6.1. Monascus Related Citrinin Risk

**Figure 4:**
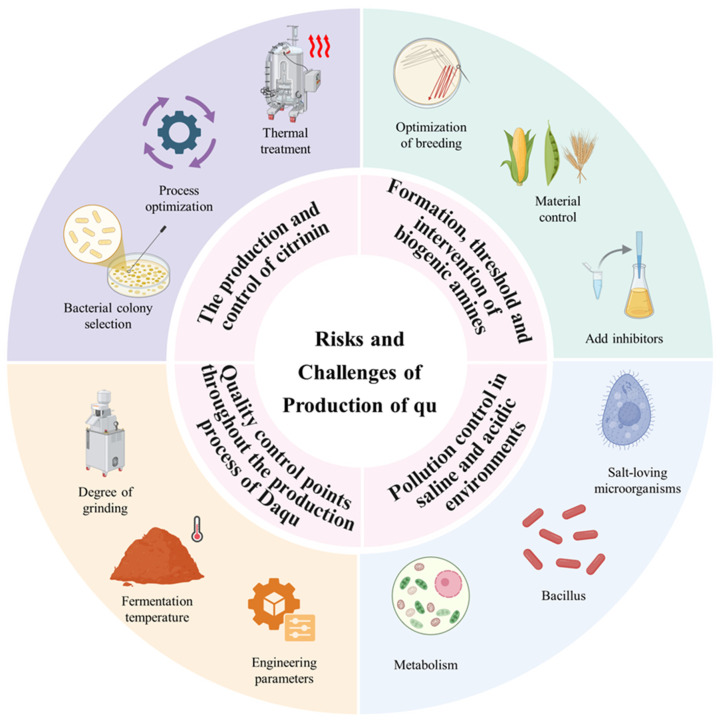
Risk identification and process control for qu.

As shown in Figure 4, *Monascus* in red koji fermentations could co-produce the mycotoxin citrinin (CIT), which is cytotoxic to kidney and liver cells [146]. CIT occurrence has been widely reported worldwide, and limits have been strictly managed. Japan set a limit of no more than 0.2 mg/kg for *Monascus* fermented products as early as 1999 [147]. For detection, HPLC was routinely used to quantify CIT with high accuracy. LC–MS/MS offered higher sensitivity and improved confirmation. Several rapid screens supported source control. On coconut milk agar under UV, *Monascus* that produced CIT showed fluorescence, which allowed removal of toxigenic strains at the seed stage.

CIT reduction strategies span strain selection, process optimization, and end treatment. Selecting low-producing or nonproducing *Monascus* strains was fundamental. Strains of the same species differed by several-fold. In angkak, the JK2 strain yielded 1.10 ± 0.021 µg/g CIT while the control FNCC 6008 reached 3.01 ± 0.072 µg/g, which significantly lowered toxin output. Genetic screening of pksCT or ctnA also identified weak producers [148]. Process optimization further suppressed CIT. Controlling fermentation near 28 °C minimized CIT, since *M. purpureus* produced the most CIT at about 35 °C in solid state or 32 °C in liquid and dropped at 28 °C [149]. Adjusting pH helped. The scheme started at pH 5.5 and raised to pH 8.5 at the end gave high pigment with lower CIT. Nutrient design also intervened in CIT biosynthesis. In liquid culture, adding the flavonoid genistein at 2 g/L lowered CIT by about 80%. La^3+^ addition promoted pigment while suppressing CIT. Prompt product separation and limiting late-stage degradation reduced accumulation [149]. Post treatments removed residual toxin. Although CIT was heat resistant, heating at 140–160 °C decomposed about 20% and converted a fraction to a non-cytotoxic H_2_ isomer. Physical adsorption and biological detox were combined in some studies. Magnetic nanoparticles adsorbed about 70% of CIT, while heat-inactivated yeast cells (121 °C) reached up to 98% removal [149].

In sum, combining strain control at the source, process regulation during fermentation, and terminal inactivation effectively lowered CIT risk in *Monascus*-fermented foods and realized a full chain path from risk identification to process control.

### 6.2. Formation, Thresholds, and Intervention for Biogenic Amines Such as Histamine and Tyramine

In protein-rich fermented foods, microbial decarboxylation of amino acids generates biogenic amines (BA) such as histamine and tyramine. Excess intake causes safety problems, including histamine poisoning from scombroid fish and hypertensive crises from tyramine, the “cheese effect” [150]. Food histamine at about 100 mg/kg was considered toxic for some consumers. Tyramine posed risk at 100–800 mg/kg. Phenethylamine showed a lower threshold near 30 mg/kg. Several jurisdictions set limits. In fish and fish products, common limits for histamine were 50–100 mg/kg. Some countries advised ≤200 mg/kg tyramine for high-risk cheeses. Although BA levels in most fermented foods stayed safe, some traditional products showed exceedance. In several Asian fermented soy foods, maximum histamine reached 2–27 times the suggested toxic dose. Tyramine and phenethylamine in some soy pastes also exceeded safe doses. Modern fermentation targeted BA risk through integrated controls from raw materials to strains and process [151,152,153,154,155,156,157].

Strain choice was critical. Producers with strong decarboxylase activity were avoided, and selected starters were used. In fermented soy foods, introducing nonproducing or BA-degrading strains reduced BA at the source. Inoculating *Bacillus subtilis*, *Bacillus licheniformis*, or *Pediococcus pentosaceus* in soy paste or douchi lowered histamine and tyramine in finished products. Selected LAB starters in meats and cheeses similarly suppressed spoilage producers and limited BA [151,154,156]. Raw material and storage control reduced free amino acid precursors and the load of BA producers. Materials were kept fresh and cold. Components rich in free amino acids were moderated. Low-temperature storage suppressed spoilage growth. For histamine-prone marine fish, rapid chilling was essential. At 5 °C for 10 d, histamine in mackerel stayed below about 50–70 mg/kg [158]. In warm conditions, histamine rose within hours and exceeded toxic thresholds [159]. Pre-fermentation handling such as chilling and permitted preservatives thus lowered the baseline for BA formation. Additives that selectively inhibited producers or enzymes also helped. Small doses of food grade agents or plant extracts reduced decarboxylase activity or growth. In a high-tyramine Korean doenjang model, adding 0.5% glycine, 0.1% sodium benzoate, or 0.1% potassium sorbate at fermentation start significantly lowered final tyramine versus control. Glycine cut tyramine by about 76.1%, and benzoate or sorbate lowered it by about 50% [150]. Nicotinic acid completely blocked tyramine in that model but was not practical at high levels [150]. Polyphenol-rich extracts also reduced BA. Rose polyphenols and spice extracts from cinnamon, star anise, or clove reduced histamine and tyramine in several fermented foods [159,160].

By using low BA starters, controlling raw materials, and applying selective inhibitors, BA contents were kept within safe thresholds. The chain ran from risk identification in high protein or high free amino acid contexts to process control by antimicrobial or anti-enzyme measures.

### 6.3. Contamination Control in Salt and Acid Tolerant Environments the Double-Edged Roles of Halophiles and Bacillus

Many traditional fermentations proceeded under high salt or low pH to suppress general contaminants. Halophiles and acid-tolerant *Bacillus* often survived and acted as a double-edged group. They could be beneficial fermenters or persistent contaminants. In high salt, extreme halophilic archaea posed typical hazards. Halobacterium grew well at above 20% salt and caused pink discoloration on salted fish or meats due to carotenoid pigments. This “pink spoilage” served as a visible indicator of halophile contamination. By contrast, some halo-tolerant species were used as functional fermenters. Tetragenococcus tolerated 15–20% salt and was applied in fish sauce and soy sauce to acidify and protect flavor under high salt [161,162,163,164,165,166,167,168]. Risk management balanced these roles. First, salt sources and purity were controlled. Solar salt could carry spores or cells of halophiles, so refining and sterilization received attention. High-purity refined salt reduced exogenous halophiles [169]. Second, process conditions favored beneficial groups. In early soy sauce moromi, halo-tolerant yeasts and LAB produced alcohol and acids quickly and suppressed spoilers. Overaeration or overheating was avoided to prevent blooms of halophiles. Color and viscosity were checked. Abnormal pink spots or slime signaled possible halophile contamination and triggered corrective actions such as removing surface layers or raising salt, though quality had already been affected [170,171,172].

*Bacillus* also played key roles in salt- and acid-tolerant systems. In many fermented soy or grain foods, *Bacillus* such as B. subtilis acted as beneficial groups. They produced proteases and exopolymers and improved flavor and functions, as in natto and douchi. Yet risk species existing in the family. B. cereus and B. thuringiensis were reported in traditional soy pastes and douchi and produced heat-stable toxins such as the emetic cereulide. Food poisoning risk rose when B. cereus exceeded about 10^5^ CFU/g [173]. Salt or acid did not fully suppress spores. B. coagulans grew near pH 4 and caused flat sour spoilage in acidic canned foods, with large economic losses [174]. Thus, *Bacillus* had to be used while guarded.

Process control introduced beneficial competitors and tightened hygiene. Inoculating dominant *Bacillus* suppressed harmful species. In low-salt cooked fermented doubanjiang, adding beneficial B. licheniformis antagonized B. cereus that had been deliberately introduced. Initial counts of 3–4 log CFU/g dropped to undetectable levels after fermentation while product quality improved and BA remained low. This showed strong antagonism in practice. Routine microbiological monitoring and sanitation were reinforced. Batches with B. cereus above limits were discarded or reprocessed. Environments were cleaned and disinfected to prevent spore buildup. In late high-salt stages or during drying, moderate heat steps such as brief dry heat at about 80 °C were considered to kill vegetative cells. Spores remained dormant under low moisture and did not grow in finished products [16,173,175,176,177].

In summary, halophiles and *Bacillus* were leveraged for their tolerance, while salt, temperature, pH, and hygiene were precisely controlled to prevent shifts from symbiosis to spoilage or pathogenicity. Problems were anticipated by risk identification and solved by process control.

### 6.4. From Experience to Standards Quality Control Points Across Raw Materials, Qu, Fermentation, and Finished Products

Traditional fermented foods had relied on experience and manual skills. Modern industry practices introduced quality control points and standardized each stage, from raw materials to qu or starters, fermentation, and finished products [15,178].

Raw material control was the start. All inputs were tested for safety and quality and pretreated when needed. For fermented vegetables and pickles, vegetables were fresh and free of pesticide residues and pathogens and were washed with clean water. In commercial production, food grade sanitation was advised, such as soaking in chlorinated water at about 100 ppm free chlorine followed by rinsing. Some studies advised a second rinse at 50 mg/L after salting to further reduce residual contaminants. These steps replaced or quantified traditional sun drying or repeated washing and were formalized as SOP. For grains, moldy kernels were removed and aflatoxins were tested to prevent hazards from entering the system (https://www.gov.mb.ca/agriculture/food-safety/education-resources/pubs/fermented-veg.pdf, accessed on 17 October 2025).

Control of qu or starter assured purity and stable function. Natural inoculation or back slopping carried risk of drift. Standardized qu or pure starters were adopted, and QCP were set in qu making. In soy sauce koji, substrate sterilization, inoculum size, and temperature and humidity were controlled. Finished koji were tested for enzyme activity and microbial load such as total molds and coliforms. Only qualified lots entered fermentation. In red koji rice, quick screens removed toxigenic strains. Coconut agar with UV retained only non-fluorescent isolates for qu, which prevented CIT producers from entering production [149].

Fermentation control was central. Environmental conditions and in-process indices were given limits and monitored in real time. In fermented vegetables, pH drops indicated safety. For kimchi, experience said that room temperature fermentation for days gave sourness. Modern work set clear CCP. At 18–22 °C, pH had to fall to 4.6 or below within 48 h to suppress pathogens. If pH did not reach 4.6 by day 3, the batch was deemed failed and unsafe and had to be discarded. A schedule also specified targets for slow low-temperature fermentation (https://www.gov.mb.ca/agriculture/food-safety/education-resources/pubs/fermented-veg.pdf, accessed on 17 October 2025). In soy sauce, salinity, moisture, and amino nitrogen were monitored against standards. About 18% salt prevented nonhalophiles, and salt was adjusted if it fell. Modern tools such as online sensors and IoT upgraded experience to data-driven control [15,178]. Continuous pH and temperature logging with cloud alarms enabled early correction and kept conditions within standard ranges. This replaced purely sensory judgment.

Finished product control closed the loop. Products were released only after meeting safety indices in sensory, physico-chemical, and microbiological tests. For raw ready-to-eat ferments, pH had to stay in the safe range and maturation time had to ensure die-off of residual pathogens. In Korea, raw kimchi with pH < 4.2 was advised to be held under refrigeration for at least two weeks so acid-tolerant pathogens such as some E. coli O_157_:H_7_ declined to safe levels before sale [179]. Red koji products were tested for functional components such as monacolin K and for CIT against regulations. Dried fermented soy foods were tested for water activity and for toxins and BA. Products carried traceable batch records and standards.

### 6.5. Embedding the Qu to Fermentation Process Within HACCP and Regulatory Frameworks

To place the main risks such as *Monascus*-related citrinin, biogenic amines, and contamination under high salt or low pH within an auditable safety system, the stages from raw and auxiliary materials through qu making, primary fermentation, and storage or release were mapped to a food safety management framework centered on HACCP and aligned with current regulations and standards. At the operational level, the Codex General Principles of Food Hygiene and its seven HACCP principles served as the backbone to establish hazard analysis, critical control points, and verification with records, while the management system requirements of ISO 22000 [180] were used to formalize procedures. For markets in the European Union or those adopting EU standards, chemical hazards such as citrinin in red yeast rice raw materials and finished-product release limits were aligned. For the United States, consistency mapping was made to hazard analysis and preventive controls under FSMA in 21 CFR Part 117 (https://en.wikipedia.org/wiki/ISO_22000?utm_source; http://data.europa.eu/eli/reg/2004/852/oj; https://ecfr.io/Title-21/Part-117, accessed on 16 October 2025).

For concrete points of risk and control, three complementary paths were distinguished. First, prerequisite programs at the source focused on gatekeeping for raw materials and strains, including screening *Monascus* strains for toxigenic potential and baseline control of freshness and contaminant load, and key parameters such as moisture, salinity, pH, and thermo-humidity trajectories were managed as operational PRPs. Second, CCPs were set at a small number of nodes with decisive impact on safety outcomes, including time control before acidity or salinity reached safety thresholds and conformity checks for heat treatment or inactivation. Third, verification and release required terminal indicators and sampling frequencies that were benchmarked to regulations; for example, the EU maximum level for citrinin in food supplements based on rice fermented with *Monascus purpureus*, and internal control lines and retain-sample retesting were set accordingly. These practices converted chemical and microbiological risks into a tabulated system of parameters, limits, and evidence (https://eur-lex.europa.eu/legal-content/EN-CS/TXT/?from=en&uri=CELEX%3A32023R0915&utm_source, accessed on 16 October 2025).

The interface with regulations and systems balanced regional differences with audit friendliness. Within the EU, food business operators established procedures based on HACCP under Regulation (EC) No 852/2004 and maintained hazard analyses and records appropriate to product and process. In the United States, the emphasis lay on preventive controls rather than universal mandatory industry HACCP, and firms documented, monitored, and corrected process, allergen, and sanitation preventive controls under 21 CFR Part 117. For multi-market operations, ISO 22000 functioned as a unifying management skeleton that aligned the technical core of Codex HACCP with organizational requirements for documented systems, internal audits, and management review, which reduced duplication in multi-pronged compliance (http://data.europa.eu/eli/reg/2004/852/oj; https://ecfr.io/Title-21/Part-117, accessed on 16 October 2025).

In sum, a matrix linking hazards, parameters, and evidence was applied across the entire process. High-priority risks including citrinin, biogenic amines, and halotolerant or spore-forming contaminants were tied to strain and raw-material admission as PRPs, to process windows as OPRPs or CCPs, and to terminal verification as release indicators and regulatory limits. Monitoring, corrective actions, verification, and recordkeeping were fixed stepwise into traceable forms and audit evidence. This path remained consistent with Codex HACCP principles and interfaced smoothly with EU 852/2004, US 21 CFR Part 117 and ISO 22000, converting risk identification into an integrated compliance solution across processes and markets.

Overall, quantitative control points across raw materials, qu, fermentation, and finished products, together with modern monitoring and automation, upgraded traditional fermentation from experience to scientific standards. Safety and consistency were improved, and a full process loop from risk identification to process control was achieved.

## 7. Conclusions and Prospects

### 7.1. Limitations and Knowledge Gaps

#### 7.1.1. Lack of Standardization in Metagenomic and Metabolomic Protocols

Metagenomic and metabolomic workflows still lack unified standards and a minimum information set for solid-state fermentation scenarios. Generic frameworks such as MIxS and STORMS did provide metadata and reporting elements, yet no reusable industry checklist had formed for key dimensions such as daqu source, heap temperature history, moisture trajectory, and sampling layers, which limited cross-study reproducibility and model transfer. On the metabolomics side, the MSI system and later reporting norms existed, but the suitability of identification confidence and cross-platform calibration remained insufficient in high-salt and high-viscosity matrices, which broke the evidence chain for data sharing and pooled analysis. Process variables and multi-omics metadata needed to be listed as required items in parallel.

#### 7.1.2. Poor Linkage Between Enzyme Activity and Sensory Outcomes

The quantitative linkage between enzyme activity and sensory evaluation remains weak. Existing work often used correlation networks or data fusion to depict associations between metabolites and sensory scores, which could suggest pathways and candidate molecules, but could not infer effect sizes in oral perception from the strength of extracellular enzyme profiles during the koji stage because of matrix effects, interactions, and supra-threshold integration. The lack of a causality-centered loop that linked enzyme profiles, key flavor factors, and sensory contribution hindered generalizable predictive models. Subsequent studies should place standardized sensory protocols and repeatable chemical quantitation in one design and replace post hoc correlations with prospective designs and intervention trials.

#### 7.1.3. Limited Modeling of Thermohydrometric Dynamics in Solid-State Fermentations

Dynamic models of temperature and humidity in solid-state fermentation still did not capture spatiotemporal gradients at the bed scale. Predictions built only on initial concentrations and temperature curves were unreliable in traditional food systems, and seasonal or process fluctuations enlarged errors. Existing heat and mass transfer models were often built under idealized beds and limited sensing, which failed to cover nonuniform aeration, local rewetting, and self-heating peak shifts in real koji rooms. Observable modeling for actual equipment was needed, with online sensing and identifiable parameters and with water activity and gas–solid coupling included as state variables, so that consistency control across seasons and sites could be supported.

### 7.2. Integration of Traditional Wisdom and Modern Biomanufacturing

#### 7.2.1. From Natural Inoculation to Targeted Functional Seed Qu: Isolation, Recombination, and Solid-State Scale-Up of Core Microbes

To meet scale and consistency, natural fall-inoculation with experiential screening moved toward functional seed qu guided by target flavor. Core microbes with key metabolic routes were isolated from mature qu or fermentation matrices. Their aroma-forming, enzyme-producing, or stress-tolerant traits were clarified. Simplified or synthetic consortia were then recombined into solid media so that saccharification, acid or alcohol formation, and esterification were co-designed, tunable, and scalable within a single seed qu. This idea was extended to integrated design across seed qu, fermented grain, and pit mud. Multi-omics with metabolic-flux quantification verified causal links between blend ratios and outcomes, enabling reproducible modules for goals such as raising esters, lowering lactic acid, enhancing umami flavors, or suppressing off-notes.

#### 7.2.2. Smart Qu Making: Digital Monitoring and Closed-Loop Control of Temperature, Humidity, Airflow, and Biogenic Heat

Solid-state qu making featured tightly coupled heat release, heat transfer, and mass transfer. Peak internal temperatures reached about 55–62 °C with relative humidity near 90 percent. If ventilation and humidification or heat removal were not synchronized, vertical gradients in temperature and moisture increased, causing local inactivation or contamination windows. Online sensing and closed-loop control of temperature, humidity, and O_2_/CO_2_ were therefore required in industrial rooms. Layered sensors across the qu bed were combined with forced air and variable-frequency fans to predictably reduce thermal gradients as airflow increased. Exhaust humidity and dew-point monitoring allowed inference of bed moisture to guide misting. Digital-twin and physics-data fusion models indicated that solid-state fermentation, including koji for soy sauce, already supported triple loops of online sensing, cloud models, and local execution for quality consistency. Evaluations of packed-bed fermenters emphasized that forced aeration played dual roles in oxygen supply and heat removal, which governed the coupling of peak temperature and moisture. Thermal imaging or fiber thermometry, exhaust spectra, and pressure-drop monitoring formed a multi-dimensional data stack that upgraded operation from experiential inspection to early anomaly detection, prediction, and correction.

#### 7.2.3. Low-Salt or Low-Sugar and Health-Oriented Products: Technical Challenges and Flavor-Compensation Strategies

Consumer demand for salt reduction and metabolic health requires new balances between lower NaCl, flavor retention, and safety. In soy sauce and related systems, benchmark salt levels were often 18–20 percent *w*/*v*, while WHO suggested daily salt intake at or below 5 g. Process options included staged salting, late salt addition, and partial replacement with KCl or potassium lactate, supported by flavor compensation. Model-food studies showed that an 18 percent sodium reduction in soups did not reduce liking, indicating that umami and aroma synergy could help maintain acceptance. KCl-based blends that claimed no added sodium increased stated acceptance. For qu-based products, a branch worth exploration enriched GABA or raised polyphenols or small peptides to offset the loss of savory flavors and body under salt reduction. Online phase plots combining texture, volatile profiles, and amino nitrogen guided tri-objective compromises among low salt, flavor, and safety. Coupling with smart qu rooms allowed co-monitoring of lactic acid and amine risks and microbial load to curb over-acidification and rises in biogenic amines during salt reduction.

#### 7.2.4. Balancing Regional Flavor and Geographical Indications

Future work would couple geographical indications with process fingerprints to balance shared identity and local character. At the shared level, internationally recognized GI frameworks would lock raw materials, processes, and environmental boundaries. Zhenjiang Xiang Cu obtained EU PGI registration in 2012 and specified brewing materials and geographic scope. Shaoxing Jiu entered the China–EU mutual recognition list, which provided a legal basis for cross-border compliance and name protection (https://eur-lex.europa.eu/legal-content/EN/TXT/HTML/?uri=CELEX%3A52020PC0214&utm_source, accessed on 16 October 2025).

At the individual level, quantifiable phenotypes from qu, substrates, and micro-environments outline flavor maps. For example, Pixian Douban in China specified sources of inputs and the spatiotemporal rhythm of sun exposure and turning, setting verifiable thresholds for sun fermentation and qu-driven aroma building. Technical specifications appeared as machine-readable metadata, which reduced drift between name and flavor. A two-layer assessment followed. GI constrained geography and inputs, while process fingerprints described flavor character through thermal trajectories in qu rooms, moisture and ventilation curves, and key physicochemical indices. This enabled benchmarking in global trade while preserving differentiated regional expression.

#### 7.2.5. Evidence Linking Qu, Foods, and Health

Health functions driven by qu needed an evidence chain from molecular mechanisms to models to human data. Future work would combine human trials with real-world evidence in foods. With explicit labels of starter type, dose, matrix, and population subtypes, interventions on lipids, blood pressure, and sensory acceptance would be deposited in open platforms to build reusable, multi-layer evidence networks that support regulatory compliance and clinical translation.

#### 7.2.6. Open Data and Standardization: Reproducible Qu Evaluation and Cross-Process Benchmarking

**Figure 5:**
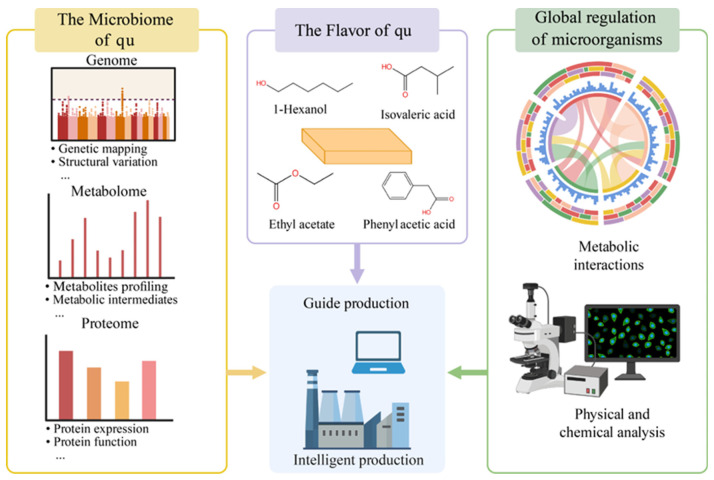
Schematic of the modernization outlook for qu.

As shown in Figure 5, upgrading craftsman experience to computable processes required open data and standardization in concert. On standards, the Regional Standard for Fermented Soybean Paste set quantitative thresholds for quality factors; for example, total nitrogen at or above 1.6 percent for soybean pastes, amino nitrogen at or above 0.3 percent, and moisture at or below 60 percent. These brought key physicochemical indices from starter to final product into unified methods under CXS 234, together with additive and contaminant limits and labeling rules, to form comparable evaluation frameworks across companies and regions (https://www.fao.org/fao-who-codexalimentarius/sh-proxy/fr/?lnk=1&url=https%253A%252F%252Fworkspace.fao.org%252Fsites%252Fcodex%252FStandards%252FCXS%2B298R-2009%252FCXS_298Re.pdf, accessed on 16 October 2025). On open data, microbial and metabolite platforms already supported FAIR practices [181,182]. Qiita enabled cross-study reanalysis and rapid meta-analysis. GNPS/ReDU and the unified cross-repository identifiers connected MetaboLights, Metabolomics Workbench, and GNPS/MassIVE for reuse of untargeted data, which informed cross-project benchmarking of qu, volatiles or peptides, and flavor. A minimum information set was therefore proposed. It recorded qu room temperature, humidity, airflow, filling and ventilation parameters, and unified fields for α-amylase and protease activities, amino nitrogen, and key volatile panels. Raw sequence and spectral files were archived, and reanalysis scripts were released together. Regional process differences were then translated into comparable process fingerprints, enabling reproducible benchmarking of starters across companies, processes, and regions.

### 7.3. Conclusions

This review restated the contemporary role of starter koji from three dimensions: process, community, and quality and safety. The key boundary conditions of solid-state starter making were summarized as moisture, oxygen supply, ventilation and heat removal, and thermal history trajectory. A reproducible process fingerprint was built for benchmarking across processes and seasons. Evidence showed that temperature type was the primary driver of daqu assembly and functional differentiation. Replacement of related phyla and genera and their metabolic directions occurred alongside heat peaks, gradual cooling, and maturation. In the soy sauce system, the salt-tolerant relay between the koji stage and the moromi stage, together with the adaptation of lactic acid bacteria and halotolerant yeasts under high salt, provided a model of cross-stage coordination between enzyme supply and salt-tolerant conversion. In the Hongqu scenario, CIT risk control and regulatory limits further indicated that quality and safety had become constraints embedded in process design. These points supported a modern redefinition of starter koji as a natural bioreactor.

Traditional experience was therefore replaced and elevated by two paths. The first was process parameterization. Online visualization and limit management brought peak temperature, heating rate, bed pressure drop, and water activity into process control points. Within the HACCP framework of fermented foods, acidification, salinity, and time–concentration trajectories of key hazards were turned into release thresholds. The second was microbiome-guided design. Metagenomic and metabolomic analyses informed the assembly of starter consortia and the optimization of symbiosis around functional cores and interaction networks, which balanced flavor and safety. The coupling of these paths constituted a shift from workshop craft to engineering and from experience to reproducible manufacturing.

Future work was suggested in three actionable directions. First, cross-platform standards for omics and process data should be formed to support long-term accumulation on community assembly rules and functional cores. Second, a closed evidence loop from enzyme profiles to material flux and to sensory and consumer responses should be established to move metrics from physicochemical endpoints to explainable sensory causality. Third, dynamic models of solid-state fermentation that couple heat and mass transfer with metabolic flux should be developed and linked with online sensing and digital twins to predict and correct batch variation. These efforts would align reproducible thermal and moisture trajectories, designable community structures, and verifiable quality and safety into a unified engineering language and would accelerate the transition of Chinese fermented foods toward standardization and high consistency.

## Figures and Tables

**Table 1:** Summary overview of microbial diversity and community ecology.

System	Ecological Driver and Key Boundaries	Dominant/Core Taxa (Genus Level)	Functional Orientation and Representative Metabolism	Typical Spatiotemporal Features
Daqu—low temperature	Lower temperature regime; mild heat stress; acidity gradually increased	*Pichia*, *Lactobacillus*, etc.	Saccharification and acid production proceeded in parallel, forming a gentle metabolic base	Early yeast dominance followed by LAB takeover; smooth succession
Daqu—medium temperature	Moderate peak temperature; spatial structure with a “more enriched center layer”	*Bacillus* (higher in the inner layer), *Saccharopolyspora* (higher in the outer layer); filamentous fungi *Aspergillus*, *Rhizopus*, *Thermomyces*, *Thermoascus* co-occurred with layer preferences	Inner layer favored primary hydrolysis of glyco-/proteinaceous substrates; surface favored thermotolerant enzymes and lipolysis jointly drove flavor precursor conversion	Community reassembly coupled to “heat peak–slow cooling–maturation”; stabilization during storage
Daqu—high temperature	Strong selection by high-temperature pulses and extended holding	*Bacillaceae*, *Kroppenstedtia*, *Desmospora* with thermotolerant fungi (e.g., *Thermoascus*); *Lentibacillus* dominated in the high-temperature window	Efficient cleavage of proteins/polysaccharides and generation of nitrogen-containing flavor precursors; pyrazine-related pathways active	α-diversity decreased then increased; functional core reshaped; Firmicutes proportion markedly increased
Xiaoqu community	Rice-based solid state with few core genera and concentrated dominance	*Rhizopus*, *Saccharomyces*, *Lactobacillus* (with *Weissella*/*Pediococcus* at the start)	Mold-led saccharification, yeast-driven alcohol fermentation; LAB established an acidic environment and coupled to ethyl lactate formation	Three-stage symbiosis: “*Rhizopus* first—yeast takeover—LAB steady state”; xiaoqu provided the core backbone while the environment supplied additional bacteria
Red koji community	*Monascus*-led and biosynthetic gene clusters (BGCs) steered metabolism; pigment/lovastatin outputs balanced against CIT risk	*Monascus*, *Saccharomyces* (with *Aspergillus* in some systems)	Pigment and polyketide synthesis; subsequent yeast and LAB co-modulated flavor; CIT was modulated by strain and interactions	Succession of “Monascus leading—yeast takeover”; pathway enrichment differed across starters
Wheat-bran/soy-sauce koji community	Two-stage ecological relay: solid-state, oxygen-rich koji → high-salt, low-oxygen moromi	Koji stage: *Aspergillus* with early colonizing bacteria; moromi stage: *Tetragenococcus*, *Staphylococcus*, *Zygosaccharomyces* rouxii and other halotolerant members	Front end built an extracellular enzyme library and depolymerized substrates; back end completed acidification and N/C-skeleton transformations at 18–22% NaCl	Fungal signals decayed rapidly in moromi; bacterial diversity converged over time; temperature–oxygen strategy shaped dominant genera
Douchi-qu community	Typified assembly across “starter—natural microbial pool—salt infiltration and ripening”	*Aspergillus*-type, *Mucor*/*Rhizopus*-type, and *bacterial*-type; *Bacillus* dominated in the bacterial type	*Aspergillus*-type relied on mold leadership with subsequent yeast/bacterial relay; bacterial type depended on a *Bacillus* backbone with osmotic-tolerant yeasts to deepen flavor	Stepwise succession; systematic differences in co-occurrence networks and metabolic emphases
Sufu mold-curd community	Short-course, high-humidity molding; mold–bacterium synergy for texture	Core molds: *Actinomucor*, *Mucor*, *Rhizopus*; later convergence of LAB and Enterococcus	High-throughput extracellular hydrolases during molding shaped “cheese-like” texture and precursor supply; during salting/ripening, bacteria took over flavor formation	Molds built the base; bacteria took over under high osmotic/low-oxygen conditions; the starter mold pool was stable across regions

## Data Availability

The original contributions presented in the study are included in the article, further inquiries can be directed to the corresponding author.

## Abbreviations

AEDA	Aroma extract dilution analysis
aw	Water activity
BA	Biogenic amines
BGC(s)	Biosynthetic gene cluster(s)
CCP(s)	Critical control point(s)
CFU	Colony-forming unit (e.g., CFU/g)
CFR	Code of Federal Regulations (e.g., 21 CFR Part 117)
CIT	Citrinin (mycotoxin)
EC	European Communities/Commission (e.g., Regulation (EC) No 852/2004)
e-nose	Electronic nose
EU	European Union
FD (factor)	Flavor dilution (factor)
FSMA	Food Safety Modernization Act
GABA	γ-Aminobutyric acid
GC–O	Gas chromatography–olfactometry
GI	Geographical indication(s)
HACCP	Hazard analysis and critical control point(s)
HPLC	High-performance liquid chromatography
HT-Daqu	High-temperature daqu
IoT	Internet of Things
ISO 22000	Food safety management standard by the International Organization for Standardization
KCl	Potassium chloride
LC–MS/MS	Liquid chromatography–tandem mass spectrometry
LT-Daqu	Low-temperature daqu
LAB	Lactic acid bacteria
MK	Monacolin K
MT-Daqu	Medium-temperature daqu
NaCl	Sodium chloride
O_2_/CO_2_	Oxygen/carbon dioxide
OAV	Odor activity value
PGI	Protected geographical indication
PRP(s)	Prerequisite program(s) (ISO 22000 context)
QCP(s)	Quality control point(s)
RH	Relative humidity
ROAV	Relative odor activity value
SOP	Standard operating procedure(s)
SP (activity)	Saccharifying power (enzyme activity)
SPME–GC–MS	Solid-phase microextraction gas chromatography–mass spectrometry
UV	Ultraviolet
WHO	World Health Organization
*w*/*v*	Weight/volume
*v*/*v*	Volume/volume
